# Defining Operational Limits of Low-Cost MSLA 3D Printing for Microfluidic Systems: Resin Performance, Cell Compatibility, and Fluid Dynamics

**DOI:** 10.1021/acsomega.6c02218

**Published:** 2026-08-12

**Authors:** Yago Radziunas-Salinas, Bárbara Blanco-Fernández, Vanessa Valdiglesias, María Teresa Flores-Arias, Carmen Bao-Varela, Ana Isabel Gómez-Varela

**Affiliations:** † Photonics4Life Research Group, Applied Physics Department, Faculty of Physics, 16780 Universidade de Santiago de Compostela 15782, Santiago de Compostela, Spain; ‡ Instituto de Materiais (iMATUS), Campus Vida, Universidade de Santiago de Compostela 15782, Santiago de Compostela, Spain; § I+D Farma Group (GI-1645), Department of Pharmacology, Pharmacy and Pharmaceutical Technology, Universidade de Santiago de Compostela, E-15782 Santiago de Compostela, Spain; ∥ Health Research Institute of Santiago de Compostela (IDIS), Complexo Hospitalario Universitario de Santiago de Compostela, Travesa da Choupana s/n., Santiago de Compostela 15706, Spain; ⊥ NanoToxGen Group, Centro Interdisciplinar de Química y Biología (CICA), Department of Biology, 16737 Universidade da Coruña 15071 A Coruña, Spain

## Abstract

Additive manufacturing using low-cost masked stereolithography technology is an attractive option to democratize microfluidic devices fabrication, albeit with limits in spatial resolution, optical transparency, cytotoxicity, and replication that need to be addressed. In this work, five transparent masked stereolithography resins employed in an Anycubic Photon M7 Pro low-cost printer are systematically studied to address the limitations mentioned above. The working route was based on the analysis of the dimensions of negative and positive microfeatures, their transmission spectra, their cytotoxicity, the capability of building internal channels, replicability in PDMS, and validation by computational fluid dynamics simulations with experimental fluid assays. Resolution tests revealed that negative structures resolved less accurately due to subtle resin accumulation during the layer-by-layer printing process. Internal channel fabrication showed a practical lower limit of approximately 500 μm under the printing and postprocessing conditions used in the study, with resin viscosity, printing angle, and drainage behavior strongly influencing channel clearance. The Standard V2 and High Clear resins exhibited the highest printing resolution with microchannels printed in a vertical or almost-vertical position. Regarding thermally cured PDMS replication, the Tough Ultra, ABS-like Pro 2, and Water-Wash were the ones exhibiting the most accurate outcome. From a biological point of view, all resins were non-cytotoxic, with Standard V2 and High Clear exhibiting the highest cell viability, in some cases better than the control scenario. Contact angle measurements indicated that the Standard V2 and High Clear resins are highly hydrophilic, whereas the remaining resins exhibit a hydrophobic behavior. Swelling was found to be dependent on the media, where the Water-Wash found high swelling in Milli-Q water and PBS and the Tough Ultra in ethanol. Samples were only minimally affected by isopropyl alcohol. Finally, fluid flow assays were conducted on three different microfluidic chips to demonstrate that the introduction of microfeatures and zigzag architectures fosters the disruption of laminar flow conditions by introducing secondary flows and vorticity to mimic more physiological environments. These experiments were contrasted with simulations performed by computational fluid dynamics. It was verified that microfeatures can be adequately implemented in the chip architecture and result in enhanced mixing. This validation allowed defining a practical operating window for MSLA 3D printers for microfluidics.

## Graphical abstract

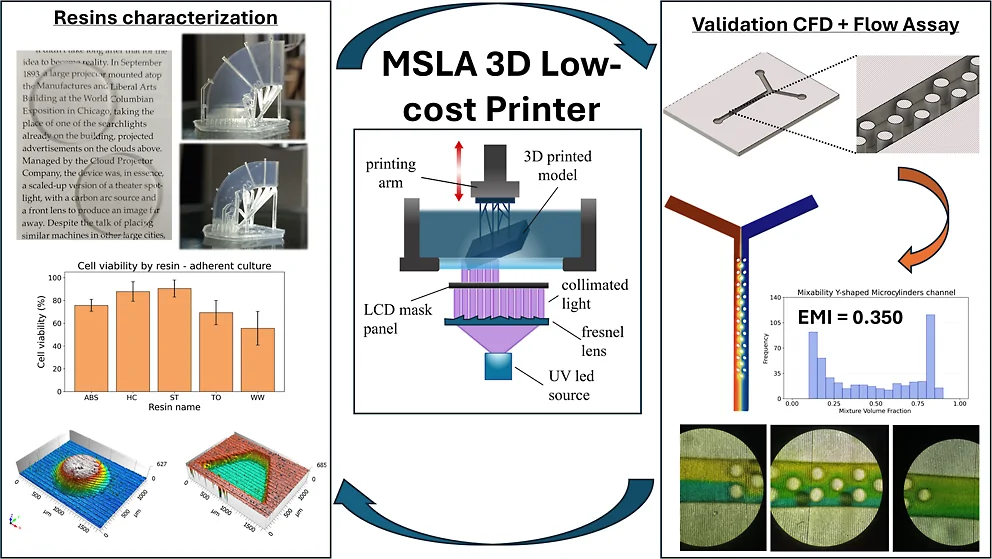

## 1 Introduction

Microfluidic technologies have revolutionized the way we engineer, analyze, and interact with fluid systems in the nano, micro, and milli scales, enabling precise control of chemical gradients, cell microenvironments, and the development of lab-on-a-chip architectures.^1–5^ Traditionally, the fabrication of microfluidic devices required highly costly working spaces, such as cleanroom facilities. The techniques employed to create these devices commonly rely on photolithography to produce a high-fidelity master in materials such as SU-8 on silicon.^6–10^ Then, a soft lithography process is followed by casting a replica material, such as polydimethylsiloxane (PDMS)^11–13^ or a thermoplastic element (e.g., Flexdym).^14–17^ While these methods result in an adequate control of microfeature resolution and correct surface quality, the requirements bound to the infrastructure, costs for the whole experimental outline, time consumption, and low eco-friendliness are drawbacks limiting rapid and safe prototyping.^18–21^

In recent years, novel techniques are seeking to overcome the limitations of the aforementioned methods. Additive manufacturing approaches have emerged as promising routes to create microfluidic platforms in a more rapid and less constrained manner. Among them, vat photopolymerization techniques such as stereolithography (SLA), digital light processing (DLP), and masked SLA (MSLA) do fulfill the requirements for a neat and flexible creation of microfluidic devices, either directly or acting as soft lithography masters.^22–24^ All of them rely on the photopolymerization principle, where a light source interacts with a liquid resin deposited in a tank, performing a layer-by-layer curing process. In the case of SLA, a laser source (normally in the UV regime) interacts directly with the resin, moving along the tank by a set of mirrors. For the DLP approach, a light projector is used as the curing agent for photoresins. The light source is projected using a series of micromirror devices laid out in a matrix, where color, brightness, and contrast can be controlled. The projector can handle each mirror individually to reflect light toward the screen on the vat or away. Finally, MSLA refers to the employment of any SLA process where the light source is partly covered or masked. In many systems (as the one in this study), LCD screens are the masks used for selecting the desired cross-section of the part. Figure 1 depicts the principles behind these three techniques.

**Figure 1:**
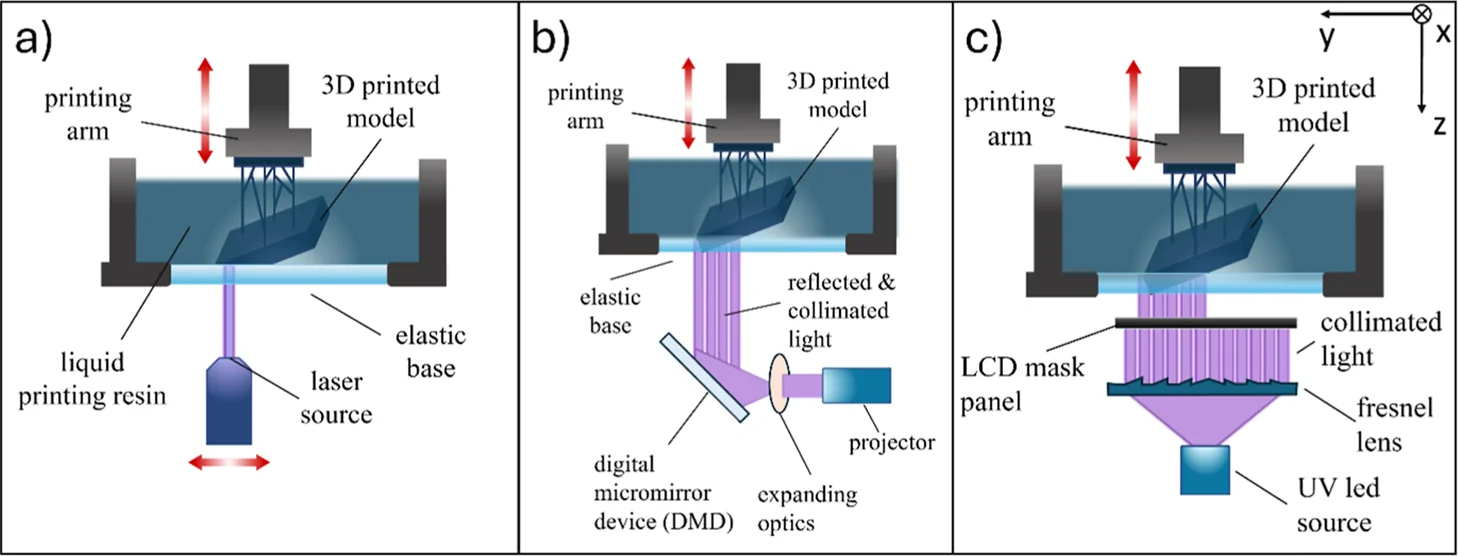
Photopolymerization techniques for 3D printing: (a) SLA, (b) DLP, and (c) MSLA.

Concerning resolution, each technique offers different crucial parameters: in the SLA approach, resolution is determined primarily by the laser spot size, the resin composition, and the step size of the stage. For DLP, this fact is linked to the size of the micromirror device, whereas for MSLA, resolution is dependent on the pixel size of the LCD screen.

These approaches enable the direct printing of all the elements of the final chip, including microfeatures in a wide range of sizes and heights, offering a very attractive pathway for democratizing the fabrication of microfluidic devices.^25,26^ In this study, special attention will be put into MSLA printers, where the implementation of high-resolution microstructures employing low-cost MSLA printers faces several challenges that limit the broad versatility.

Spatial resolution and feature fidelity remain limiting factors in the printing process. Although conventional MSLA printers typically operate in the range of the pixel, there are other determinants to the resolution related to the morphological characteristics of the chip. Particularly, the response in resolution terms is biased by the presence of negative or positive structures, being affected by different parameters. Positive features are characterized by the exposure size, whereas negative ones are limited by the minimal separation at the edge of two exposure points.^27,28^ Although some studies do offer a deep study of the surface resolution or internal channel printing by performing test structures,^29–31^ there are few works in the community analyzing systematically multiple resins and microfeatures to discover the resolution limit within these low-cost systems.^23^

Optical transparency and light transmission are other crucial elements for the microfluidic realm. In many applications within microfluidics, such as the mimicking of biological structures (namely organ-on-a-chip technologies), microscopy and fluorescent assays are usual techniques for characterization.^31–33^ Resins for high-resolution patterning incorporate absorbers for the photopolymerization processes along with photoblockers and quenchers,^34,35^ hindering the employment of fluorescent dyes in the spectrum the material absorbs or introducing visualization difficulties such as autofluorescence. These conditioners limit the final extent of the microfluidic chips directly created with resins, thus making relevant the characterization of the optical properties of the photoelements. A third element, representing a bottleneck in the development of cutting-edge applications within organ-on-a-chip approaches, is the cytotoxicity and biocompatibility of the resins.^36–38^ A comprehensive comparison among different resins is key to encouraging the direct employment of these devices for applications where cell seeding is necessary.

Finally, replication capacity into other materials keeps being a highly valuable characteristic for photoreagents, acting as soft lithography molding systems. However, PDMS curing may have difficulty when interacting with the resin surfaces. The presence of photoreactors and unreacted components leaching from the resin causes a poisoning of the PDMS catalyst, a process called PDMS cure inhibition.^39^ This issue is present among 3D-printed resins,^40,41^ and further post-treatment strategies should be studied to enable a clean molding process.

In this work, we investigate the achievable spatial resolution of affordable MSLA printers with five transparent resins in the visible spectrum, systematically exploring both positive and negative surface features. We quantify the minimum resolvable dimensions in positive and negative relief, evaluate the optical transmission and clarity of each resin, and assess the cytotoxicity under controlled conditions. Furthermore, we demonstrate the fabrication of internal microchannels in these materials and investigate the replication fidelity in PDMS. Wettability tests were also conducted by measuring the contact angle and swelling of the five resins in different media. Finally, we provide an experimental validation by fabricating microfluidic chips and assessing their mixing performance via both computational fluid dynamics (CFD) simulations and experimental flow assays. Crucially, our approach emphasizes affordability: the hardware (a desktop MSLA printer) and resins used are accessible to many academic laboratories, and yet we show that they can approach performance levels relevant for sophisticated microfluidic research.

By systematically evaluating the interplay among geometry, optics, biocompatibility, and replication fidelity, we intend to chart a more rigorous and practical path toward low-cost, high-resolution microfluidics using masked stereolithography. Our results not only demonstrate feasibility but also provide guidelines and benchmarks for researchers seeking to adopt affordable MSLA-based techniques in microfluidics, organ-on-chip, or related lab-on-a-chip applications.

## 2 Materials and Methods

### 2.1 3D Masked Stereolithography Printer

All samples were fabricated using the Anycubic Photon Mono M7 Pro (Shenzhen, Guangdong, China), a high-resolution MSLA (Masked Stereolithography Apparatus) printer operating with a 405 nm UV light source. The novelty of the system in comparison with the conventional SLA printing technology lies on the monochrome 14K LCD panel (13.312 × 5.120 pixels) to selectively polymerize photosensitive resin in a layer-by-layer approach, achieving a resolution within tens of microns on the *XY* plane. The system possesses a flexible sheet acting as a base, reducing the side stresses of the final piece.

Besides, the printer incorporates the Anycubic’s LighTurbo 3.0 optical engine, which includes a high-intensity UV LED matrix coupled with a Fresnel lens collimation system. This optical setup ensures uniform light distribution and minimal angular distortion (lower than 3°), producing collimated, perpendicularly incident UV beams across the entire printing surface. The Fresnel lens, in particular, reduces optical path divergence and mitigates edge-related exposure inconsistencies (parallax), assuring a uniformity above 90% on the LCD panel.

To check the resolution of the printer, several microstructures were designed and printed. By means of the Fusion 360 CAD software (San Francisco, USA, version 2604.0.316), plates of 3 × 4 cm (XY dimensions) containing negative and positive cones, trapezoids, and rectangular semichannels were created. The shape of the structures is shown in Figure 2a,c.

**Figure 2:**
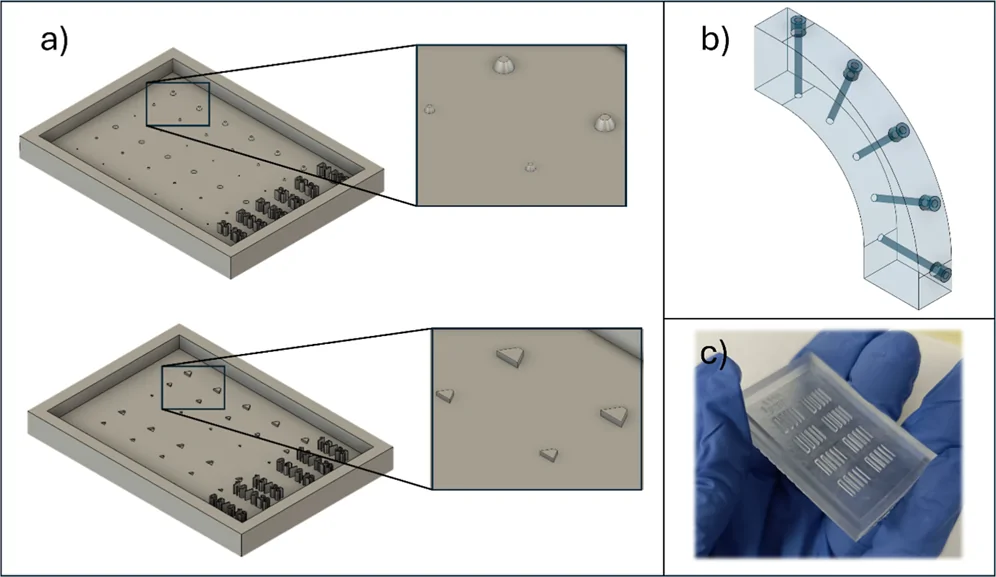
To achieve the resolution limit of the Anycubic Photon Mono M7 Pro, different sets of negative and positive (a) cones and trapezoids, (b) internal microchannels, and (c) lines were printed.

Cones featured varying diameters (from 125 to 1000 μm) and heights (from 125 to 500 μm), trapezoids showcased different edge sizes (from 120 to 1500 μm), and rectangular channels featured different widths (from 150 to 1000 μm). All samples were printed in a negative and a positive configuration.

To investigate the possibility of printing internal round channels, tests with varying diameters (from 250 to 1000 μm) and angles (a set of 5 configurations between 0° and 90°) were conducted, as performed by previous authors.^31,42^ The shape of the samples for this angle-dependent study is shown in Figure 2b.

All samples were postcured after the printing process. Resins (except the Water-Wash resin) were washed in an isopropanol bath (>90°) with active agitation for 10 min employing the Wash & Cure 3 Plus Station (Shenzhen, Guangdong, China). For the Water-Wash resin, a washing with deionized water was conducted for 10 min with the Wash & Cure 3 Plus Station. Then, all samples were UV-cured for an additional 8 min process with the 405 nm lamp using the Cure 3 Station.

To carry out the biocompatibility testing, different experiments were conducted in both adherent and suspended cell cultures. On one hand, discs with 3 mm height and 3 cm diameter were divided into 1 cm × 1 cm × 3 mm (length × width × height) pieces to perform direct cytotoxicity assays with adult human dermal fibroblasts (NHDFs) as a model of adherent culture. On the other hand, discs featuring holes of 25 mm × 2 mm size were printed to conduct a circulating cell cytotoxicity assessment, employing in this case a human monocyte cell line (THP-1) that was seeded in each disc and analyzed 72 h after seeding.

### 2.2 Materials

Five transparent MSLA resins from Anycubic were evaluated: Water-Wash Resin+, Standard Resin V2, High Clear Resin, ABS-Like Resin Pro 2, and Tough Resin Ultra to assess their optical transparency and performance for microfluidic applications. Recommended printing parameters were selected for all the resins and samples, featuring a 50 μm *z*-height layer resolution. Table S1 in the Supporting Information lists these parameters for the Anycubic Photon Mono M7 Pro printer within the resins.

For replication, the masters were cast in PDMS, a material widely used for microfluidic applications.^43–45^ To deepen the assessment of the replication capability of the resins, two distinct PDMS variants were employed. First, thermally cured PDMS (one of the most widely used materials for lab-on-a-chip applications) was prepared using 184 Sylgard Elastomer (Dow Chemical Company, Midland, Michigan) by mixing the monomer and the cross-linking agent in a 10:1 ratio, as reported in the literature.^46,47^ Once both components were mixed, the resulting polymer was introduced into a vacuum chamber for 40 min at 400 mbar to remove the air bubbles from the mixing process. Masters were infilled with the bubble-free PDMS and placed in an oven at 60 °C for 12 h until solidification was accomplished.

Second, ultraviolet-curable PDMS was prepared from KER-4690 A/B (Shin-Etsu Europe BV). It consists of two components, KER-4690 A and KER-4690 B, that the manufacturer recommends mixing in a 50:50 weight ratio. The mixture was degassed in the same conditions as before and cured by irradiation with a 405 nm LED at 5.34 mW/cm^2^ for 20 min in a chamber heated at 50 °C (FormCure, Formlabs Somerville, Massachusetts, USA).^48^

### 2.3 Cell Culture and Cytocompatibility Assessment

To broaden the applicability of low-cost resins in microfluidics, especially in organ-on-a-chip platforms, the cytocompatibility was assayed in NHDFs. NHDFs (Lonza, Catalog #: CC-2511) were cultured in Advanced DMEM (ThermoFisher Scientific, #12491015), supplemented with 1% GlutaMAX (ThermoFisher Scientific, #35050061), 1% antibiotic-antimycotic (ThermoFisher Scientific, #15240062), and 10% fetal bovine serum (FBS, ThermoFisher Scientific, #10270106). Cells were used at passage 5–6. NHDFs were seeded on a 24-well plate at a density of 50,000 cells/well in 0.5 mL of complete cell medium and cultured overnight to enable cell attachment. The photoresin samples (*n* = 4) were then placed inside the wells and incubated for another 24 h. The cytocompatibility was determined by measuring the cells’ metabolic activity with AlamarBlue (ThermoFisher Scientific, #DAL1025) according to the manufacturer’s instructions. Briefly, the cell medium was replaced with AlamarBlue (10% v/v in cell medium) and incubated for 1 h in the cell incubator. Then, fluorescence intensity was measured using a spectrophotometer plate reader at 560 nm excitation and 590 nm emission. Cells cultured with cell medium under the same conditions were used as a negative control.

On the other hand, a circulating cell cytotoxicity assessment was also carried out on human monocytes (THP-1, ATCC, Manassas, VA, USA, TIB-202). Cells were cultured as a single cell suspension in RPMI 1640 (ThermoFisher Scientific, #52400025) containing 15% FBS, 1% l-glutamine (ThermoFisher Scientific, #25030081), and 1% penicillin (ThermoFisher Scientific, #15070063). 30,000 cells in 0.5 mL of complete cell medium were seeded in each resin type employing 6-well plates as support. To delve into the effect of surface modification on cellular fate, oxygen plasma treatment (Diener Zepto plasma cleaner, Diener Electronics, Ebhausen, Germany) was performed for 60 seconds on each resin type. This resulted in a plasma-treated and a nonplasma-treated configuration for each resin. THP-1 cells seeded directly on the 6-well plates were employed as negative controls. All conditions were tested in triplicate. Cells were then cultured for 72 h at 37 °C with 5% CO_2_ and a humidified atmosphere. Afterward, morphological analysis and cell count were carried out with a light microscope (Nikon TMS, Japan), and phase-contrast photographs of control and resin-seeded cells were obtained in each case (Figure S11, Supporting Information).

### 2.4 Microfluidic Platform Testing

Fluid dynamics play a pivotal role in microfluidics for a wide range of applications, such as nanoparticle synthesis for drug delivery, sensor optimization, chemical analysis, cell sorting, and organ-on-a-chip systems. In this case, we deepened our understanding of the applicability of the resins to create high-performance mixers. For this aim, two different approaches were followed.

Computational fluid dynamics (CFD) simulations were performed to analyze the mixing efficiency of 3 distinct microfluidic channels: A Y-shaped channel, a Y zigzagged, and a Y-shaped channel with micropillars along the main branch. In all cases, the inlets 1 and 2 were set with a velocity of *v* = 0.001 m/s corresponding to a flux rate of *Q* = 0.078 mL/min. The outlet was set to atmospheric pressure (101325 Pa), and the remaining surfaces were set as walls with nonslip conditions. A combination of hexagonal and tetrahedral mesh was employed near the walls to enhance accuracy in regions characterized by high gradients of velocity.

Prior to testing the mixability of the channels, mesh convergence analysis was carried out. The Y-shaped channel was chosen to perform this test. Different sets of grid density were established (from 5851 to 1457277 elements), and the maximum value of velocity in all the configurations was calculated, as listed in Table 1. It was found that a grid featuring an element base size of 0.1 mm (with 116451 elements) yielded a deviation in the maximum velocity of approximately 1.1% compared to the densest grid. This configuration showcased an adequate accuracy while requiring less computational effort, being the one chosen to perform the CFD studies.

**Table 1:** Mesh Convergence Analysis for the CFD Approach

element base size (mm)	number of elements	maximum velocity (m/s)	percentual difference (%)
1.000	5851	0.003590	7.47
0.500	6197	0.003547	8.58
0.250	16654	0.003796	2.16
0.100	116451	0.003838	1.08
0.075	212988	0.003837	1.11
0.050	493877	0.003862	0.46
0.030	1457877	0.003880	0.00

Consequently, all simulations were conducted under a 0.1 mm element base size framework. Simulations were run under an implicit unsteady framework with laminar segregated flow. The time step was set to Δ*t* = 0.1 *s* with a second-order temporal discretization. The SIMPLE method was chosen, and simulations were run for 50 s, where a residual convergence of 1 × 10^–5^ in continuity was achieved. The mixing performance was tested by implementing a passive scalar model with an under-relaxation factor of 0.9. The volume fraction of the mixing species was set to 1.0 in inlet 1 and 0.0 in inlet 2.

On the other hand, experimental validation of the mixability of the chips was performed. For that purpose, a four-channel Minipulse 3 peristaltic pump (Gilson, Middleton, Wisconsin, USA) was used, ensuring consistent flow conditions for accurate measurements and observations. To demonstrate the mixability of the chips, two different color dyes (blue and yellow) were introduced simultaneously into the system. This method enables not only visualizing but also quantifying the mixing performance of the chip, as the combination of both dyes results in a distinct third color. The flow was established through a 3-stop pump platinum-cured silicone tubing (inner diameter (ID) 1 mm and outer diameter (OD) 3 mm) (Darwin, Paris, France), connected with stopper connectors (BFlow SL, Santiago de Compostela, Spain) and silicone tubing (ID 1 mm and OD 3 mm). Straight connectors (BFlow SL, Santiago de Compostela, Spain) were used to introduce the flow in the micromixer.

### 2.5 Data Collection

Confocal images of the microstructures and superficial roughness measurements were obtained using a 3D optical profilometer S neox (Sensofar Metrology, Terrassa, Spain). A Nikon MM-400 metallurgical microscope (Nikon Instruments Europe B.V., Amsterdam, The Netherlands) was used for the internal channel and PDMS replica evaluation, given the out-of-range depth of the profilometer. The mixing performance was also evaluated by means of the Nikon MM-400 metallurgical microscope, analyzing the resulting color after the mixing of both dyes. To measure the transmission spectra of the transparent resins, a PerkinElmer Lamb25 spectrometer (PerkinElmer Inc., Waltham, Massachusetts) was employed. This spectrometer measured the transmittance of the sample disks between 190 and 1100 nm in 1 nm intervals.

Regarding the cytotoxicity of the samples, adherent assays were performed in quadruplicates, whereas suspension culture studies were conducted in triplicate. Experimental data were expressed as mean ± standard error.

Contact angle measurements were conducted on the surface from the five resins. For that aim, discs of 3 mm height, 30 mm diameter (*n* = 3) were printed under the 45° configuration, and then their surface static apparent contact angle θ_*c*_ was measured by a Phoenix MT­(A) Analyzer. Two representative polar liquids were chosen to conduct this study: Milli-Q water and glycerol.

Swelling analysis was conducted for the five MSLA resins. For that, discs of 3 mm height and 30 mm diameter were divided into quarters and introduced (*n* = 3) in 4 different liquids: Milli-Q water, PBS (phosphate-buffered saline), isopropanol 95%, and absolute ethanol. Masses of the samples were measured two times prior to their introduction in liquids and then after 1 h, 1 day, and 5 days. The relationship between the mass at time *t* = 0, and the times mentioned before enables the swelling percentage to be obtained as follows

1 $$\%\;Swelling=\frac{\left(m_{t=X}-m_{t=0}\right)}{m_{t=0}}\times 100$$

where *X* corresponds to 1 h, 1 day, or 5 days, respectively.

## 3 Results and Discussion

### 3.1 Printing Time

A pivotal element in 3D printing frameworks is the printing speed, especially when evaluating their implementation on an industrial basis. For that reason, the time employed for printing the set of elements described in Figure 2 (see Figure S1 in the Supporting Information) was evaluated for each of the resins. This allowed for a direct comparison among the resins, as seen in Table 2.

**Table 2:** Printing Parameters for the Pieces Used in This Study

resin name	printing time (min)	volume (ml)	*V*/*t* (μL/min)
ABS-like Pro 2	79	57.5	727.8
High Clear	104	57.5	552.9
Standard V2	72	57.5	787.8
Tough Ultra	94	57.5	611.7
Water-Wash+	93	57.5	618.3

The Standard V2 resin was found to be the fastest resin, with a maximum *V*/*t* value of 787.8 μL/min, followed by the ABS Pro and Water-Wash+ resins. These results are in accordance with the slice parameters proposed by the Photon Anycubic Software in the Supporting Information (Table S1), where the Standard V2 resins exhibit the lowest normal exposure time (1.8 s) and off time (0.5 s). On the other hand, the High Clear Resin was found to be the one needing a higher time (104 min). This fact is due to its normal exposure time (4.0 s), the highest in comparison with the other resins. Concerning the printing prices (Table S1), the most expensive approach is working with the High Clear and Tough Ultra resins. The prices for the remaining resins are similar.

### 3.2 Resin Transmission Spectrum

With the willingness to apply these low-cost 3D printing systems in lab-on-a-chip applications in the microfluidics realm, optical transparency emerges as a key point. Optical inspection is necessary to assess the performance of the microfluidic system. In addition, most of these systems rely on characterization techniques such as confocal microscopy or fluorescence, where transparency is vital to evaluating the elements in the chip as well as the signals from the fluorophores.

The spectrophotometer PerkinElmer 25 was employed to evaluate the response of the transparent resins under different wavelengths, resulting in the five transmission spectra depicted in Figure 3a. Discs were directly printed on the building platform to maximize transparency, as showcased in the visual inspection in Figure 3b. In other printing angles, transparency decreased due to the irregularities arising from the scaffolding supporting system (as shown in Figure S1), which scattered light more thoroughly.

**Figure 3:**
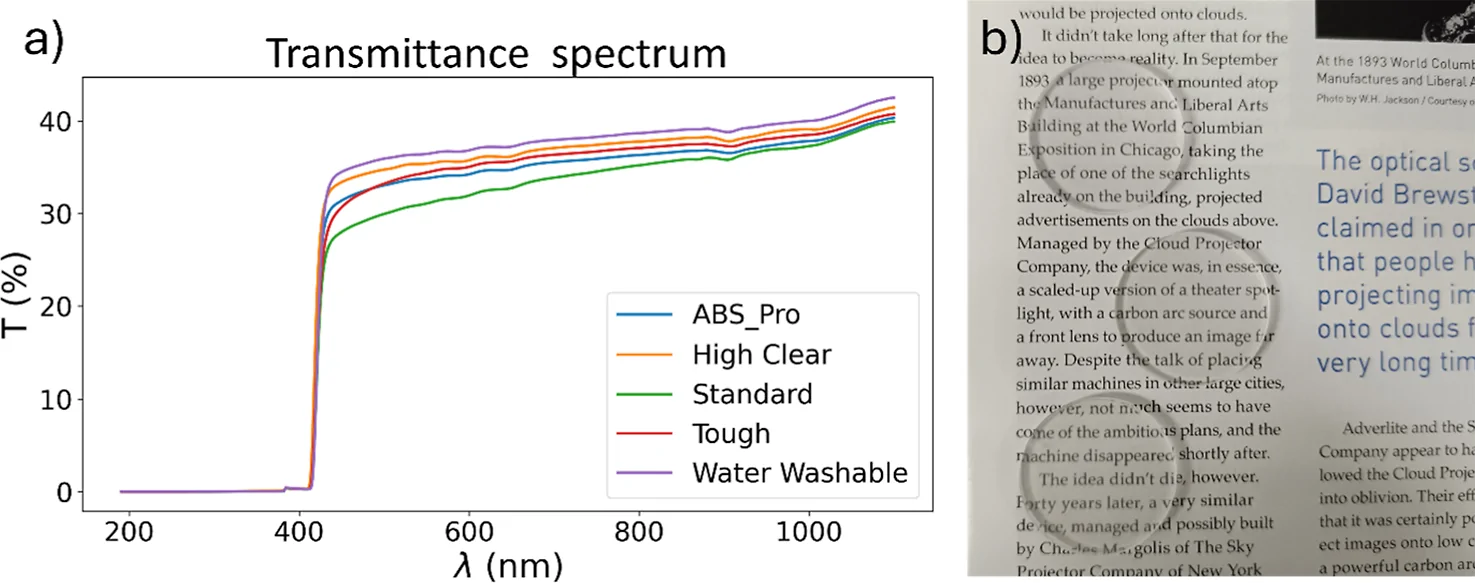
Evaluation of the optical transparency of Anycubic resins. A spectrophotometer was employed to determine the (a) transmission spectra from the resins, compared with their (b) direct optical inspection (3 Water-Wash+ resin discs).

As depicted in Figure 3a, all resins exhibit a similar behavior regarding their transmission. The highest transmission was found in the Water-Wash+ resin (42.51%), followed by the High Clear (41.47%) and Tough Ultra resin (40.75%). The resin with the lowest transmission corresponded to the Standard resin (39.91%). All resins exhibit a major decrease in transmission when wavelengths are less than 405 nm, according to the photopolymerization wavelength. Photoinitiators in the resins react to photons with wavelengths lower (energies above) than 405 nm, absorbing them and, thus, blocking their transmission throughout the sample. This dropout in the UV region of the spectrum establishes a threshold for visualization and characterization in microfluidic devices built from these resins. In many cell culture assays performed within this field, confocal fluorescence microscopy tools rely on the absorption of fluorophores. The excitation wavelength must be above the photopolymerization threshold to obtain an emission signal. Fluorescent dyes such as Alexa Fluor or rhodamine are within the high-transmission regions, whereas others, such as DAPI, are found in this close-to-zero area.

The similarities in the transmission spectra are also exhibited in the mean roughness measurements presented in Table 3. In general, all samples exhibit roughness close to a value of 3 μm, independent of the size of the region of interest. We do assume that transmission can be enhanced by means of optical polishing equipment, as conducted in similar works;^31^ nonetheless, as the systems depicted a correct transparency by visual inspection (Figure 3b), this approach will be followed for the microfluidic mixing testing.

**Table 3:** Maximum Transmission From the Transparent Resins, along with the Value of Mean Roughness for 3 Sample Sizes

resin	maximum transmission (%)	mean roughness 1 × 1 mm (μm)	mean roughness 0.5 × 0.5 mm (μm)	mean roughness 0.25 × 0.25 mm (μm)
ABS-like Pro 2	40.33(03)	3.67(17)	3.48(89)	2.80(43)
High Clear	41.47(05)	3.80(88)	4.9(2.1)	3.33(86)
Standard V2	39.91(03)	3.60(34)	3.37(39)	3.27(37)
Tough Ultra	40.75(02)	3.54(69)	3.91(59)	3.15(46)
Water-Wash+	42.51(05)	3.95(52)	3.93(54)	3.77(25)

### 3.3 Microstructures on the Surface

The role of microstructures in microfluidic devices is fundamental to broaden the application of the field. Systems such as micromixers^11^ rely on microfeatures to break the usual laminar flow found in these frameworks (low Reynolds numbers), forcing the parallel layers of fluid to recirculate and mix, creating a more chaotic and turbulent scenario. In other contexts, microstructures enable us to perform the isolation of species in many-component systems by adapting their geometrical features to the needs of the species to be separated.^49^ Finally, in branches such as organ-on-a-chips, microstructures are widely used to mimic biological structures in a precise manner. Elements such as the brain–blood barrier^50,51^ or microtumoral environments^52,53^ are cutting-edge implementations where micrometer-size elements play a key role.

A study in resolution for different microstructure geometries was performed in this work. For this purpose, 3 different elements were printed, as depicted in Figure 4a,b. Trapezoids are characterized by major (*L*_2_) and minor (*L*_1_) sides with height (*H*), whereas lines exhibit a width (*W*) and height (*H*). Height in the lines was always set to 1 mm. In the case of cones, major (*D*_1_) and minor (*D*_2_) diameters separated by a height (*H*) are disposed. During this study, condition *D*_2_ = *H* was established.

**Figure 4:**
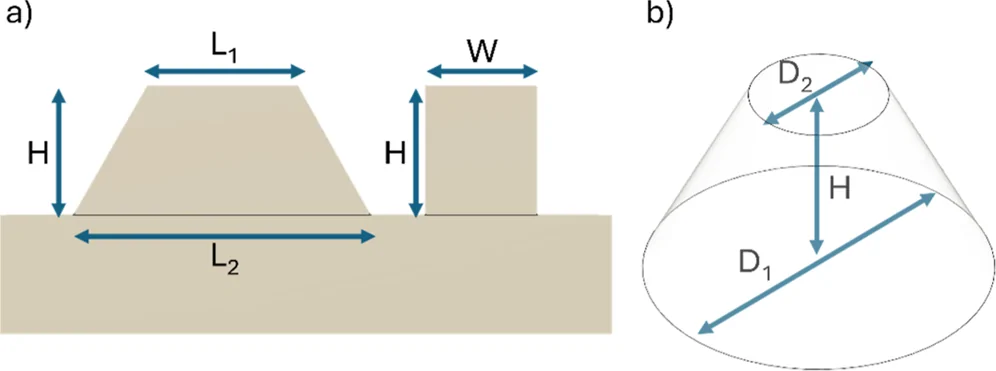
Representation of the main design parameters of (a) trapezoids, lines, and (b) cones.

Samples were characterized by means of confocal microscopy, obtaining 3D reconstructions, as depicted in Figure 5. Regarding conical structures (Figure 5a,d), a noticeable fact was discovered. Positive cones exhibited diameters for upper and lower bases similar to the ones designed in the CAD design; nonetheless, negative cones under 500 μm showed noticeable differences between the desired and the printed diameters. In general, *D*_1_ was found to be greater than 250 μm in all cases, reaching in some cases up to 300 μm (as showcased in the Supplementary Material Figure S2). Regarding *D*_2_, smaller diameters than the theoretical ones were obtained, yielding sizes close to 80 μm instead of 125 μm. This tendency was found in all resins, demonstrating the complexity of printing negative structures within the micrometric range. We hypothesize this behavior is due to the 45° angle of the pieces while being printed, where one of the two sides is most exposed to resin sliding. This sliding may be the cause of subtle resin accumulation, resulting in a minor diameter of the smaller side of the cones.

**Figure 5:**
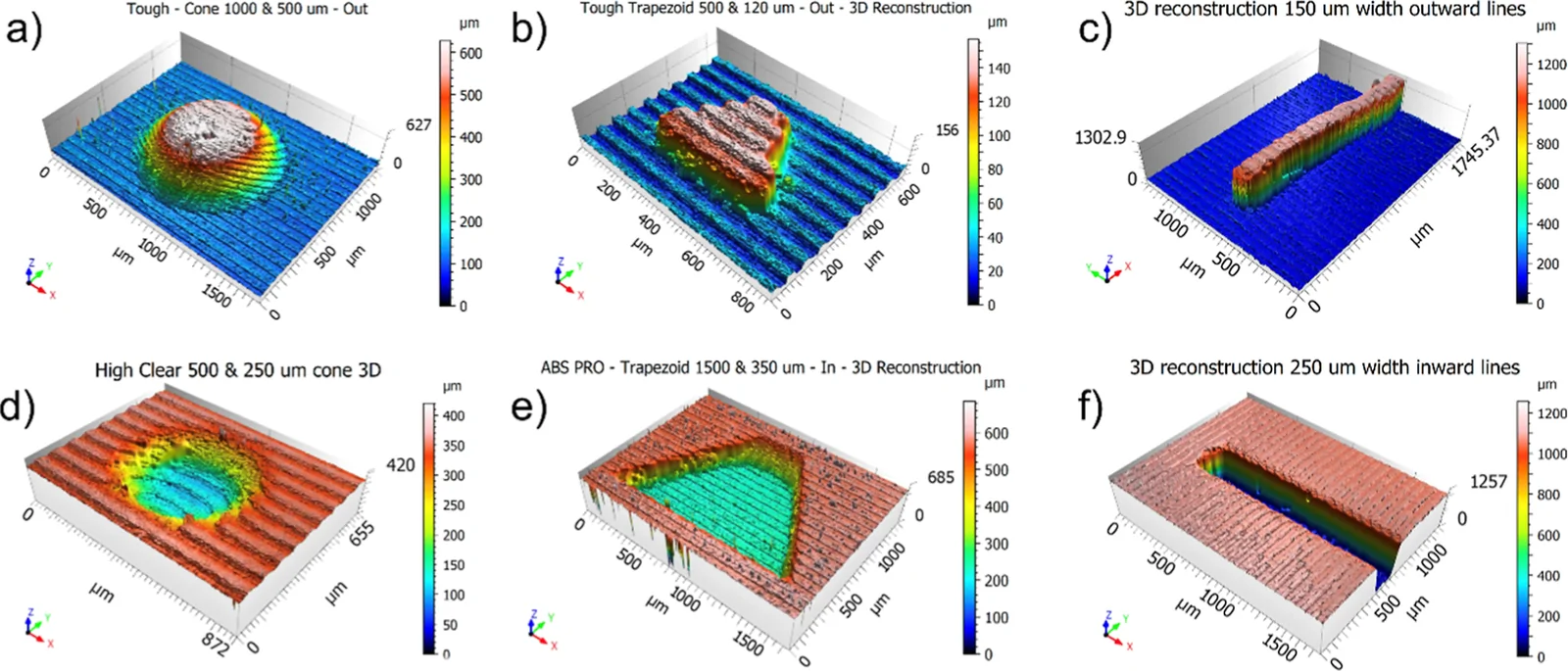
Confocal 3D reconstruction of positive (row above) and negative (row below) structures. Different sets of (a,d) cones, (b,e) trapezoids, and (c,f) lines were characterized.

Regarding trapezoidal microfeatures, an excess of resin is found close to their minor side (Figure 5e), thus turning the samples to exhibit a lower height H value. This effect can be once again attributed to the printing angle during the stereolithography process. After one layer is printed, the remaining resin slides along the L_1_ side, fostering the accumulation of overcured resin and consequently enlarging that side. It is noticeable that woodgrain structures appear along the surfaces, which is especially relevant in Figure 5b. Profile measurements indicate these features are 10–20 μm in width and are due to the Z-stage movement during photopolymerization when pieces are not printed parallel to the base. This surface microtopography can be suitable in 3D-printed microfluidics due to the relevancy of parameters such as surface roughness and wetting. Systematic studies on DLP/SLA printers show that printing conditions can measurably change surface finish and contact-angle measurements, which eventually affects capillary-driven motion and filling behavior in microchannels.^54^ Besides, droplet-microfluidics guidance treats surface roughness and native wettability as key criteria for a robust and reproducible droplet generation.^55^ Finally, work on additively manufactured microchannels highlights that tuning printing conditions can translate into changes in flow behavior and stability, reinforcing the need to report these 10–20 μm wall features when benchmarking lab-on-a-chip platforms.^56^ Finally, regarding positive and negative lines, proper results were obtained in all configurations, demonstrating the versatility of the printer. Nevertheless, in high aspect ratio cases (for instance, the 150 μm width), a bending behavior along the line was found. This happened in the Water-Wash + (Figure 5c) and ABS-like Pro 2 resins. Another valuable aspect to mention is related to the profiles of the samples. In general, especially in the negative configuration, lines do not showcase a rectangular profile but a Gaussian one. This phenomenon is due to the layer-by-layer printing process, which fosters small clogging and over-curing of resins on the walls of the feature, thus transforming the rough rectangular shape into a softer,rounder one. This tendency is depicted in the profiles in Figure S2 to S7 in the Supporting Information.

To further investigate the final dimensions of the features for all resins and facilitate the interpretation of the data, different aspect ratios for the structures are depicted in Figure 6. The chosen ratios were compared to the theoretical values from the CAD design. In the case of cones, a ratio among $\frac{D_{2th}\times D_{1th}\times H_{th}}{D_{2\exp}\times D_{1\exp}\times H_{\exp}}$ was chosen, where the subscript “th” represents the theoretical (CAD) measure and the subscript “exp” refers to the experimental one. For trapezoids, knowing the ratio between the major and minor sides (4.3 times), the reference chosen was $\frac{L_{2\exp}-3.3L_{1\exp}}{H_{\exp}}$ . Finally, for the line configuration, a ratio between width and height was established $\frac{W_{th}{\times H}_{th}}{W_{\exp}\times H_{\exp}}$ . In all cases, the ideal value for the ratios is 1. The more the deviation from that value, the higher the differences between theoretical and experimental outcomes.

**Figure 6:**
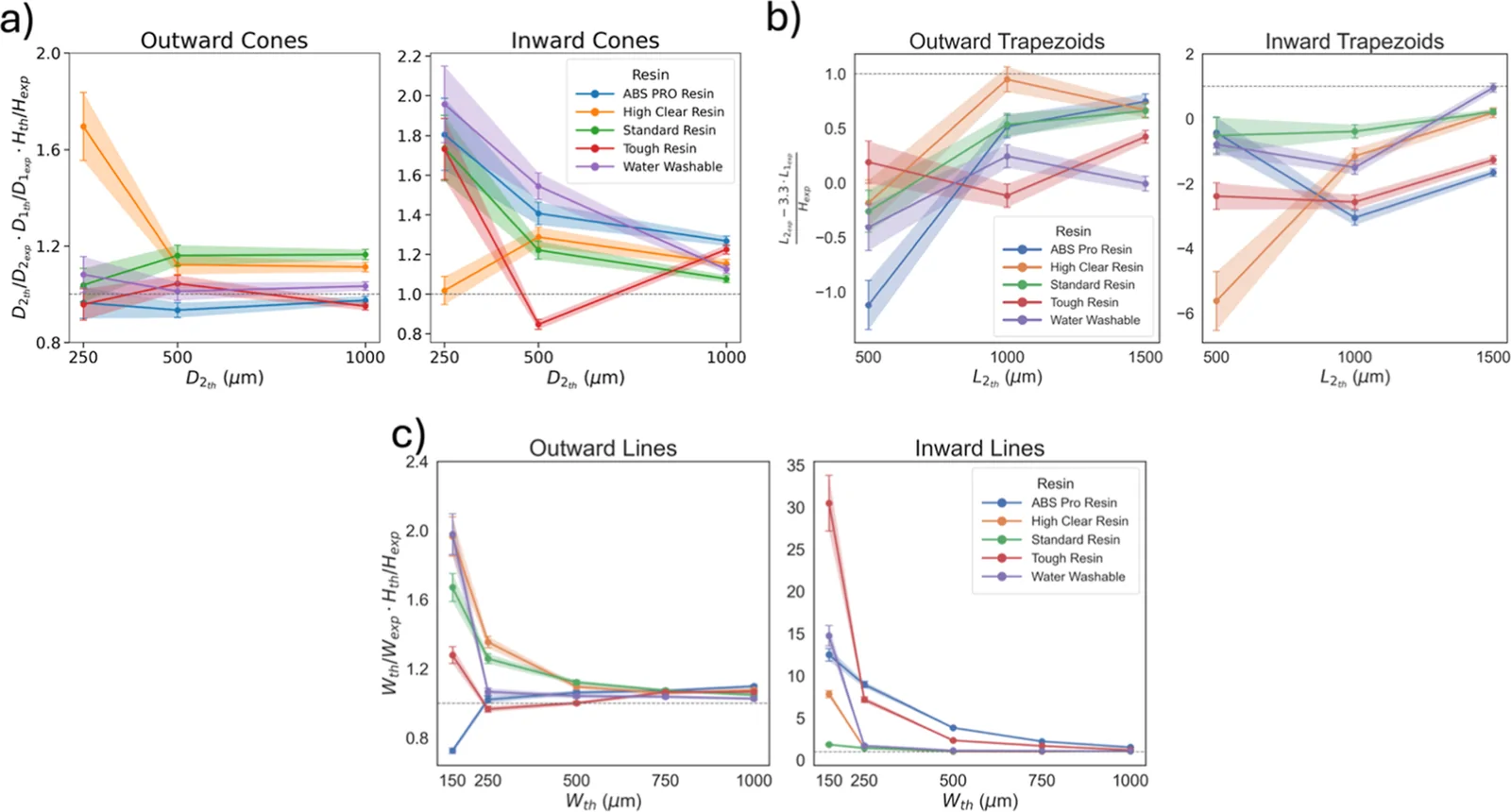
Aspect ratios from the microstructures printed on five different resins with the Anycubic M7 Pro. Studies were performed on both positive and negative configurations, focusing on the dimensions from (a) cones, (b) trapezoids, and (c) lines. Inside the graphs, each color corresponds to one of the five resins employed in this study.

Regarding positive cones, most of the values obtained are similar to the theoretical expectations. The High Clear resin is the only one exhibiting a value much higher than its counterparts, with a ratio of 1.6 for the 250 μm *D*_2_ diameter. On the other hand, the ratios for negative cones are slightly higher than their counterpart. The Water-Wash + resin exhibits the highest ratio (close to 2), and the higher the diameters, the closer to 1 are the ratios found. This tendency can be attributed to the partial clogging of resin around the inner diameter. The smaller the diameter, the easier it is for the resin to get trapped inside the microfeature, thus exposing a larger area to be cured. This phenomenon can be seen in Figure 5d, where the north region of the cone is clogged with an excess of material. In this case, not only the size of the microcone plays a pivotal role but also the printing angle.

Moving to the trapezoids in a positive configuration, we obtain a clear tendency. When moving to lower *L*_1_ and *L*_2_ sides, negative ratios are obtained (for example, for the ABS Pro resin). This phenomenon exhibits that there is probably an excess of curation on the minor *L*_1_ side, thus turning that side to be larger than the theoretical expectation. Once the dimensions of both sides are enlarged, the effects become less relevant, approaching the ratio of 1. Nonetheless, resins such as the water-washable one or the Tough resin keep having low ratios. Regarding negative microtrapezoids, similar tendencies are present. In this case, the High Clear resin is the one depicting the most negative ratio, showcasing an excessive *L*_1_ side size. On the other hand, resins such as the Standard depict a ratio close to 0 in all of the sizes.

Finally, concerning the line configurations, several aspects should be remarked. In general, positive lines exhibit a neater outcome than negative ones. The biggest ratios are obtained by resins such as the Water-Wash +or the High Clear resin. Nonetheless, once the width of the line increases, the ratio yields the theoretical value of 1. In all cases, heights are close to the theoretical 1 mm, yet the widths tend to vary. In the most critical cases (Water-Wash + or High Clear resin), the experimental width is half that of its theoretical counterpart, showcasing an underestimation of the dimensions. This is very relevant when introducing microfeatures with a stated periodicity, especially when the distance among microstructures needs to be consistent. Under these circumstances, it would be desirable to scale the dimensions of the CAD design so that the final dimensions after the printing process fit with the desired parameters.

With regard to the negative configurations, the ratios are much higher than their positive counterparts. This is due to the low heights and widths in comparison with the theoretical value of 1 mm, resulting in ratios higher than 30 in resins such as the Tough resin. This effect takes place when the printing base is immersed in the tank, where the small width of the line hinders the resin from being removed. The resin is clogged inside the rectangular profile, inhibiting the release of material.

In summary, negative configurations exhibit a higher aspect ratio in comparison to their positive counterparts. This phenomenon is not noticeable with structures within hundreds of micrometers, although the smaller the features, the greater the discrepancy. This effect takes place in both cones and trapezoids, and it is due (partially) to an excess of resin accumulation . Limitations of the resin to slide and the angle of printing do play a key role in this effect. Angles closer to zero could avoid the sliding effect of the resin and overcuring in one of the sides, although in that case, capillary forces emerge as crucial for removing the excess of resin in the negative structure. These forces are dependent on several parameters such as the viscosity of the resin, its density, and the leaving force from the platform. According to the set of parameters, the gravity force cannot be strong enough to overcome the capillary forces, hampering the evacuation of material from the negative microstructure. The limits established by these parameters are beyond the scope of this study.

Additionally, stability from the morphology of the lines was found to be dependent on the ratio between the width and height of the line. Some of the lines exhibiting a 125 μm width could not keep their straight firmness along the structure, resulting in a twisting microfeature. This behavior was found in the Water-Wash+ resin, whose chemical composition is notably different in comparison with its isopropanol-washing counterparts.

### 3.4 Internal Channels

To study the suitability of the resins to construct microfluidic devices, the capacity of printing internal channels was evaluated. One of the biggest challenges for this kind of printer is preventing partial polymerization and resin trapping within the lumen, which can lead to clogging. These issues stem from the layer-by-layer photopolymerization process, as each new layer must be mechanically supported by previously cured material, and unsupported regions are prone to collapse. Consequently, supports are often required to ensure structural integrity; however, in enclosed microchannels, such supports are difficult to remove and can compromise channel functionality. Therefore, printing internal channels involves a trade-off between maximizing channel openness while maintaining mechanical stability without introducing nonremovable internal supports.

To evaluate that, several quarter annuli structures were printed for each resin featuring channels with different diameters and angles, as proposed by other authors.^31,42^ Sizes ranged from 250 to 1000 μm, as showcased in Figure 7. As depicted there, channels in the range of 1000 μm are accurately performed for almost all angles. The only angles where clogging was found were 0° and 22.5°. In cases such as the Standard V2 resin, all the internal microchannels were successfully printed. In general, sizes above 1 mm in diameter exhibit a high degree of correct performance.

**Figure 7:**
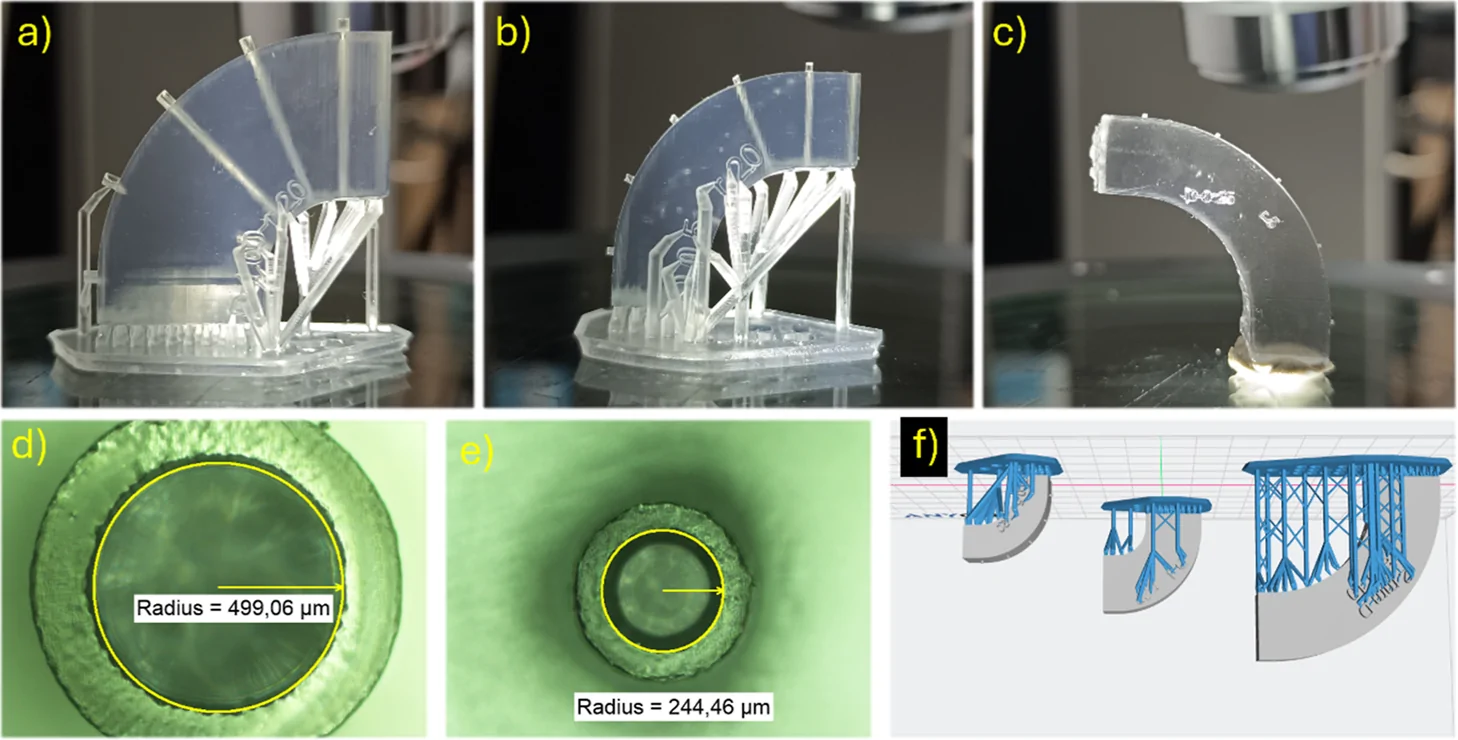
Internal microchannels according to the channel diameter and printing angle. Sizes of the channels range from (a) 1000 μm, (b) 500 μm, and (c) 250 μm. Examples with the High Clear resin. Optical microscopy images were performed to assess the inlet of the channels in the configuration of (d) 500 and (e) 250 μm. The disposal of the elements is (f) parallel to the base.

In the 500 μm size, the number of unobstructed structures diminishes. In the case of the High Clear resin, for instance, only the channels for the 90° and the 67.5° were correctly printed. These results extended to the other resins, in some cases showcasing the worst results. Resins such as Tough were able to form proper microchannels only in the 90° orientation. This result is in accordance with the different viscosities of the resins. The Tough resin is the one exhibiting major viscosity, thus increasing the drainage time over the successive layers. This effect may result in a partial or total clogging of resin in the microchannels, even in angles close to 90°.

Finally, with the 250 μm-wide channels, none of the resins nor the angles were able to depict a successful microchannel. Under this scenario, there are two physical variables playing a key role in the success of the system. Capillary pressure (ΔP_cap_) holding the resin inside the microchannel depends on the radius of the microchannel by means of

2 $$\Delta P_{cap}=\frac{2\times \gamma \times \cos\theta }{r}$$

where γ is the surface tension between the two fluids and θ is the contact angle of the wetting fluid on the solid surface. Under this case, the reduction in the diameter enhances the difference in pressure with the atmospheric one. When the pressure difference exceeds the gravitational contribution, drainage does not occur. Moreover, even when drainage is feasible, the drainage time increases as the channel radius decreases. The drainage time (*t*) is related to the flow rate according to

3 $$t∼\frac{V}{Q}$$

where *V* is the volume of the channel and *Q* is the flow rate. As the microchannel has a circular section and the resin can be considered to be a Newtonian fluid in laminar conditions, Poiseuille flow rate^57^ is satisfied

4 $$Q=\frac{\pi \times r^{4}}{8\times \mu }\times \frac{\Delta p}{L}$$

where *r* is the microchannel radius, μ is the dynamic viscosity, Δ*p* is the pressure drop between the inlet and outlet, and L is the characteristic length of the system (height of the channel in this case). As *V* ∼ *r* ^2^ × *L*, it is possible to infer that time depends on

5 $$t∼\frac{r^{2}\times L}{\pi \times r^{4}}\times \frac{L}{\Delta p}∼\frac{1}{r^{2}}$$

Therefore, the lower the diameter, the longer the time for the resin to leave the channel. This dependence of drainage time and capillary pressure on the radius may explain the clogging found in the 250 μm channels. Finally, in addition to the aforementioned conditions, there are optical phenomena playing a decisive role, such as minor laser dispersion and excessive curing depth, which could enhance an overcuring of resin, reducing even more the diameter and hindering the release of material.

Finally, to assess the translation of these internal channel simplified geometries toward a real-case scenario, a microfluidic chip resolution test was conducted. For that aim, microchannels of varying widths (1000, 750, 600, and 500 μm) were printed in the Standard V2 resin, as it was one of the materials depicting better performance in the internal channel resolution tests. Chips were printed at several angles, in accordance with the setting of the quarter annuli structures of Figure 7b. The results, depicted in Figures S8 and S9 of the Supporting Information, showed that the 90° configuration was the best-performing one. Chips with a diameter of 471(23) μm were successfully 3D-printed, yielding a reliable lab-on-a-chip device working in the absence of leakages for more than 30 min. This is to say, the simplified geometry analysis can be transferable to a functional microfluidic chip.

In conclusion, a resolution limit for internal microfluidic channels exists for the Anycubic M7 Pro 3D printer. Channels above 500 μm can be successfully printed with all resins, although the ones with a higher viscosity exhibited poorer performance. In all cases, channels of 250 μm could not be printed.

### 3.5 Replication in Polydimethylsiloxane (PDMS)

Once analyzed the versatility of the resins and printers to create microfeatures and internal channels, is valuable to evaluate their performance as soft lithography masters. Within this technique, a mold is created in the resin, acting as the negative piece for a replica. In this study, pieces were baked in an oven for 12 h at 60 °C to release any gaseous remanent which could be inside the mold. An additional UV exposure was performed for 4 h to cure any residues, as conducted by other authors,^39^ to avoid PDMS curing inhibition by the release of uncured monomers, oligomers, and compounds from the 3D-printed molds.

As mentioned in the materials section, two different PDMS variants were used for this replication study. On the one hand, the thermally cured PDMS was assessed, resulting in Figure 8. For the five resins, two of them (High Clear and Standard) could not replicate thermally cured PDMS successfully. After curing in the oven for 12 h at 60 °C, PDMS depicted a semiliquid sticky state, which was strongly fixed to the resin surface.

**Figure 8:**
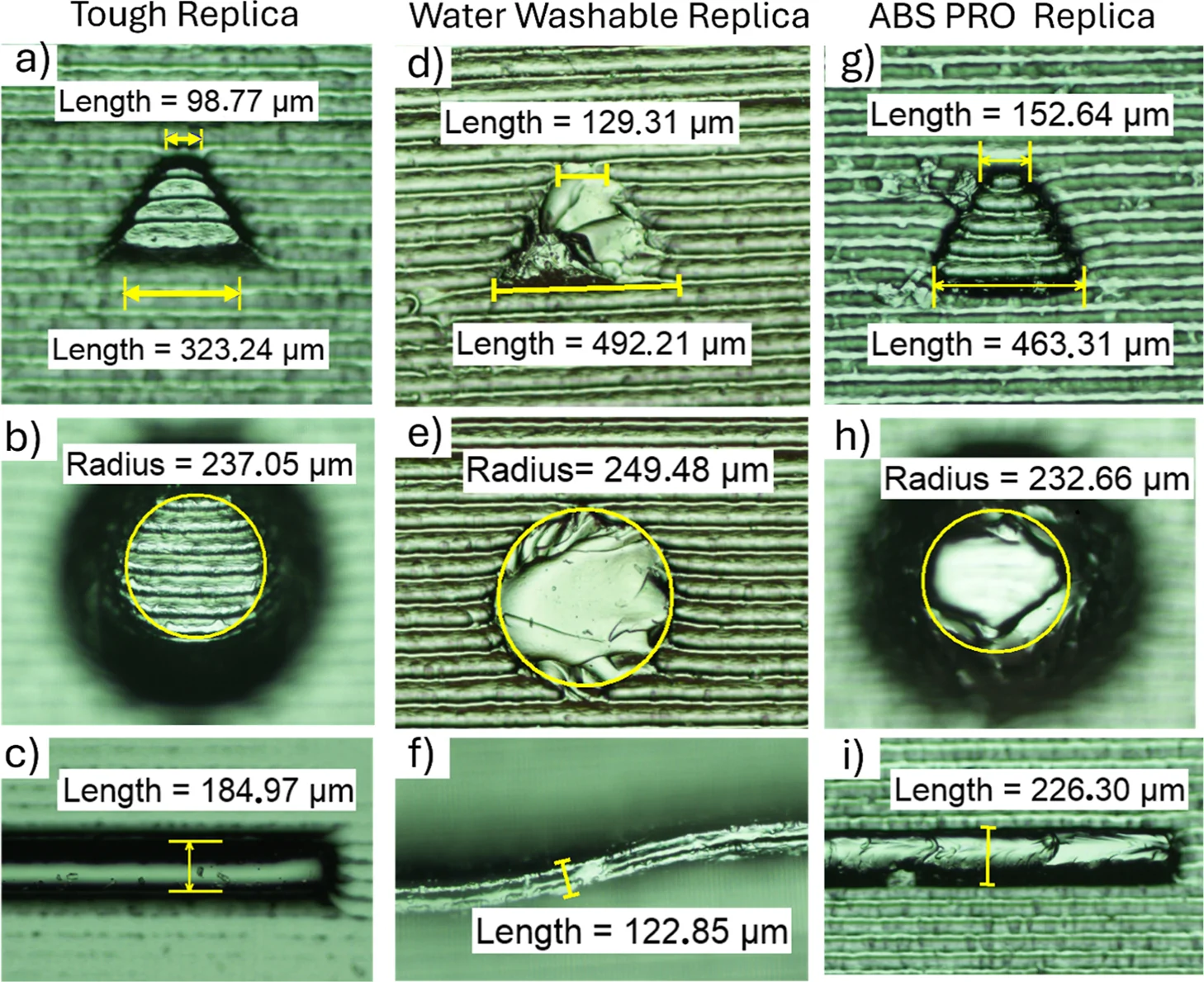
PDMS replica from microfeatures for 3 different resins. (a–c)­Tough resin, (d–f) Water-Washable, and (g–i) ABS Pro.

Nonetheless, PDMS got totally cured in the 3 remaining resins (Tough, Water-Wash +, and ABS Pro), albeit the performance in the replication was subtly different. For instance, the PDMS showcased a strong adhesion to the mold in Water-Wash +, turning the removal from the mold complex. This excess of fixation resulted in the presence of more cracks and a coarser surface, yielding a less correct replication (Figure 8d,e). The lack of straightness already seen in this resin during confocal inspection (Figure 5c) was successfully replicated in the PDMS (Figure 8f).

The ABS PRO resin removal was found to be easier than the prior case, yet with some limitations. Although the layer-by-layer stripes are correctly replicated in the case of microtrapezoids (Figure 8g), features such as the cones or the lines exhibit a wrinkled surface (Figure 8h).

Finally, the best replication was obtained with the Tough Resin. In all cases, PDMS replicates the 50 μm *z*-axis stripes from the layer-by-layer printing process. The surfaces on the PDMS exhibited roughness values similar to that of the molding replica.

However, when employing UV-curable PDMS, different results were obtained. The ABS PRO resin was able to replicate adequately the microfeatures with correct performance. Tough Ultra and High Clear depicted correct replication yet with strong adhesion, yielding to the appearance of cracks on the surface and uncured areas on the surface. Other resins (Water-Wash + and Standard V2) showcased a semiliquid state on most of the surface.

In conclusion, thermally cured PDMS was able to be replicated in 3 out of the 5 resins, with different degrees of success. The best resin for a correct and faithful replication was the Tough resin, followed by the ABS PRO and Water-Wash +, respectively. These outcomes open the door to developing molding systems for soft lithography with inexpensive approaches and resins, democratizing the advancements in the field of microfluidics.

UV-curable PDMS depicted a different performance, where only the ABS PRO resin showcased a proper replication. This phenomenon depicts the influence of photoabsorbers with the chemical components of the resin; the PDMS cure inhibition is highly dependent on the presence of photoreactors and unreacted components in the resin, and this interaction results in hampering the curation of the elastomer. Further studies should be accomplished to this extent to comprehend the mechanisms behind the proper curing process of PDMS when there are photoabsorbers on both the resin and elastomer itself.

### 3.6 Cytotoxicity Assessment

The next step in characterization relates to the biocompatibility of the resins to verify their possible application as an organ-on-chip platform. For this aim, two distinct assays were performed.

In the first of them, NHDFs were incubated with the resin samples, and the metabolic activity of the cells was evaluated after 24 h (Figure 9a). All resins showcased viability above 50%, proving their efficacy as platforms where cells can be seeded. It is noteworthy to mention the notorious viability of the Standard and High Clear resins, with values above 80%, being the most valuable resins to perform cell studies.

**Figure 9:**
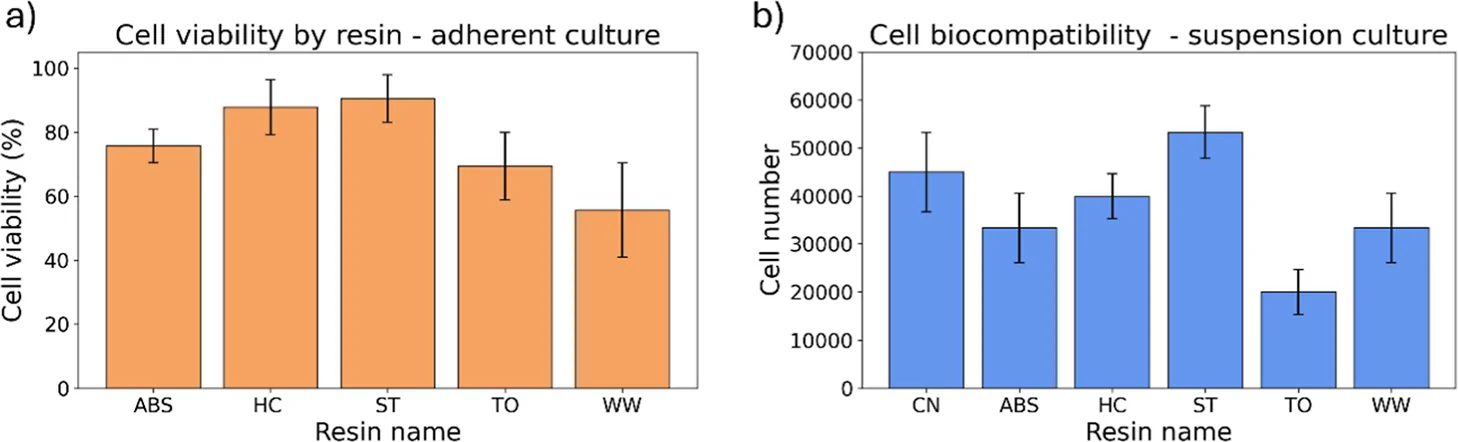
Cell cytotoxicity assays in (a) adherent and (b) suspension conditions (CN: control, ABS: ABS pro resin, HC: High Clear resin, ST: Standard V2 Resin, TO: Tough Ultra resin, and WW: Water-Wash + resin). In adherent conditions, cell viability percentages are obtained by comparison with the control scenario.

On the other hand, the potential biocompatibility of the different resins on cell suspension cultures was assessed by employing circulating THP-1 cells. Results obtained from these evaluations are shown in Figure 9b. Similar to NHDFs, the resin samples were biocompatible, as cultures were established and maintained in all resins. Nonetheless, noticeable differences in the number of cells in suspension were found. After 72 h, THP-1 cells seeded on the High Clear resins showed a similar growth to the negative control. Standard resins were found to slightly improve the cell growth, whereas cells on ABS Pro, WW, and, particularly, Tough showed a decreased growth regarding the controls. These results support the suitability of the Standard and High Clear resins to be explored for further biological applications.

To try to improve the biocompatibility of these polymeric materials (as shown in several studies^58–60^ ), a surface plasma treatment (see Section 2.3) was performed in all types of resins, and the experiments were repeated with these plasma-treated resins. Morphological analysis and cell count of THP-1 cells cultured on treated discs were again carried out 72 h after seeding and compared with the proper control. Results are shown in Figure S10 and indicate that plasma treatment improved the biocompatibility of ABS Pro, Water-Wash +, and Tough resins; however, it did not result in efficient growth for Standard and High Clear resins, which showed better growth rates in control, nontreated resins (Figure S12 in the Supporting Information). The comparison between the plasma-treated and nonplasma-treated resins is encompassed for all resins in Figure S11 by means of contrast optical microscopy images.

### 3.7 Contact Angle and Swelling Assays

To complement the dimensional, optical, and fluidic characterization of the printed devices, the surface wettability and liquid-induced stability of the five MSLA resins were evaluated. These properties are relevant as the operational performance of microfluidic devices does not depend only on the nominal printing resolution but also on the interaction between the cured resin and the working liquid. Apparent contact angles measured with Milli-Q water and glycerol were used as comparative liquids of resin wettability and time-dependent wetting behavior. These media were chosen as representative polar liquids for medical applications, as shown in different studies.^61,62^

Static contact angle values were recorded as a function of time, as the apparent contact angle of a droplet may evolve after deposition, particularly on polymeric, rough, absorbing, or chemically heterogeneous surfaces.^63,64^ Therefore, a single contact-angle value measured at an arbitrary time point may not fully describe the wetting behavior of the printed resin. Time-resolved measurements provide additional information on droplet spreading, contact-line pinning or depinning, liquid uptake, surface swelling, and possible surface rearrangement.

Since the contact angle can evolve over time depending on the material, the static contact angle (θ_*c*_) was determined 1 min after droplet deposition. Table 4 accounts for the mean contact angle with its Standard deviation for the 5 resins in both water and glycerol. It is possible to infer that the Tough Ultra, ABS-like Pro 2, and Water-Wash + resins are the ones exhibiting the highest hydrophobic behavior. Conversely, the Standard V2 and the High Clear resins are the ones closest to a hydrophilic behavior. Numerical differences were found between the two liquids (for instance, the Standard V2 contact angle changes from 49.7° to 35.3°), yet the tendencies in terms of wetting behavior remain the same.

**Table 4:** Contact Angle Measurement for the Five MSLA Resins in Two Polar Liquids

resin	liquid	contact angle θ_*c*_ (°)
Standard V2	Milli-Q water	49.7(1.2)
Water-Wash +	Milli-Q water	74.3(1.2)
High Clear	Milli-Q water	53.8(2.0)
Tough Ultra	Milli-Q water	88.2(2.0)
ABS-like Pro 2	Milli-Q water	85.4(5.3)
Standard V2	glycerol	35.3(2.7)
Water-Wash	glycerol	70.30(95)
High Clear	glycerol	55.9(2.1)
Tough Ultra	glycerol	87.03(67)
ABS-like Pro 2	glycerol	62.7(2.4)

It is also noteworthy to study the evolution of the contact angle in time, as shown in Figure 10. Some resin discs, such as the Tough Ultra or the ABS-like Pro 2, showed nearly constant contact-angle values over the acquisition time, whereas others, such as the Standard V2 and the High Clear resin, exhibited a progressive decrease up to 30° in 4 min. A stable angle suggests that, after initial droplet relaxation, the liquid–solid–air contact line reached a metastable configuration with limited spreading and limited surface rearrangement during the measuring time. In contrast, a strong decrease in apparent contact angle may indicate time-dependent wetting processes such as filling of surface microtexture, capillary penetration into microvoids or interlayer features, or progressive replacement of trapped air by liquid in rough surface features.

**Figure 10:**
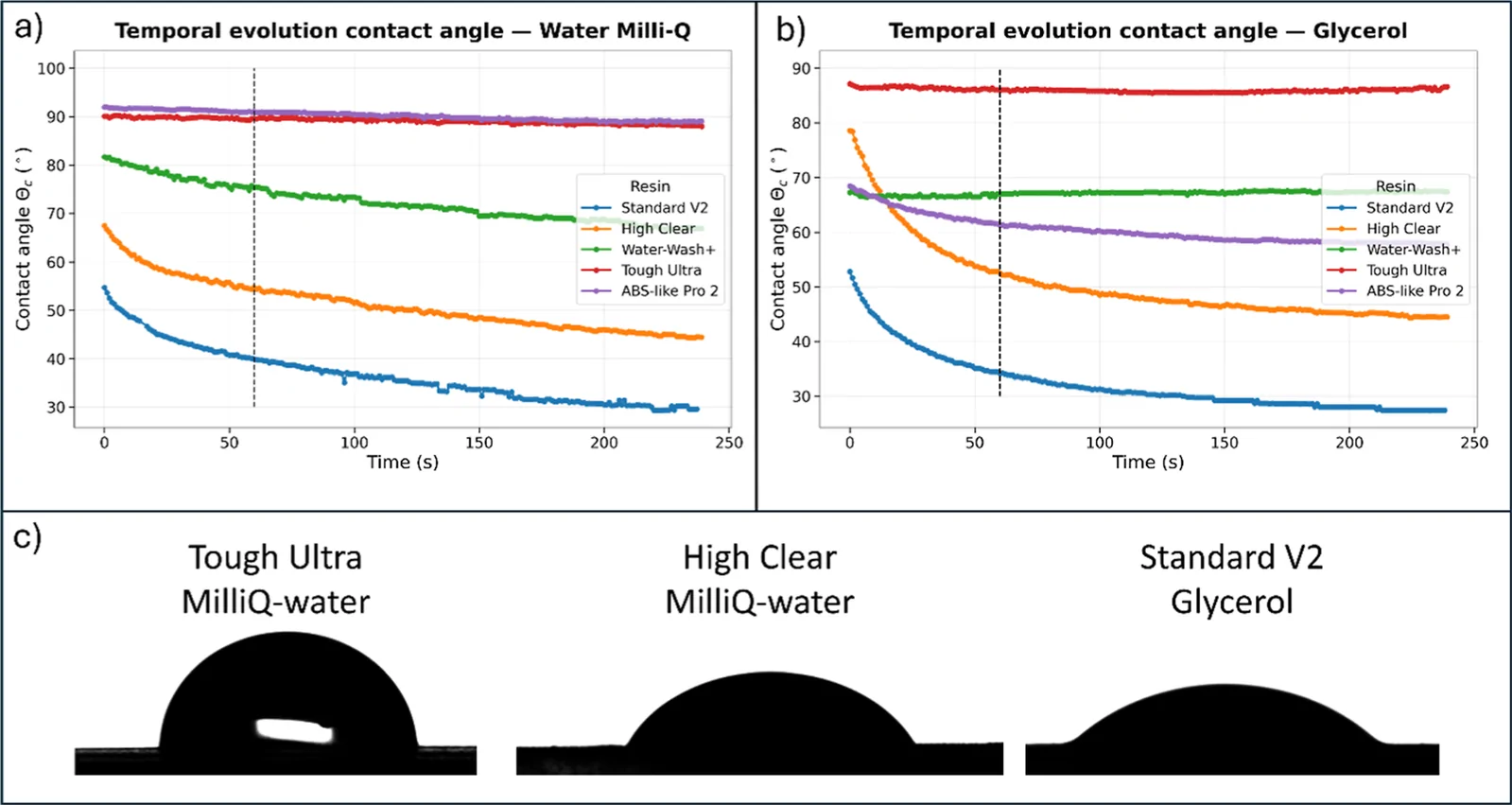
Contact angle measurements in five resins over time for (a) Milli-Q water and (b) glycerol. (c) Drop shape after 1 min indicates the hydrophobic or hydrophilic behavior of the resin.

These results are consistent with the cell assays performed using discs fabricated from the five resins, which showed that the Standard V2 and High Clear resins exhibited the highest cell viability and proliferation. Indeed, those resins are the ones exhibiting lower contact angles, aligned toward a hydrophilic nature, suitable for cell adhesion. Studies in the literature state that hydrophilic surfaces (20–40°) are the ones maximizing cell attachment in cell lines such as NIH 3T3 fibroblast and HUVEC.^65,66^ This fact, combined with other elements such as the surface chemistry and protein adsorption capability, could explain why differences in cell count were found among resins. Besides, the high contact angle in the Tough and ABS-like Pro 2 resins could be related to the low cell viability found in the cytotoxicity assessment. As previously stated in Section 3.5, surface plasma treatment improved the biocompatibility of ABS Pro, Water-Wash +, and Tough Ultra resins, essentially being the ones exhibiting the highest θ_*c*_. The reduction in the contact angle due to the plasma treatment may be responsible for the enhanced adhesion, cell count, and, eventually, compatibility.

Swelling assays were additionally conducted to determine whether liquid exposure produces measurable changes in mass, dimensions, or visual appearance. This is important for microfluidic devices fabricated close to the lower channel-size limit, since polymer swelling, solvent uptake, or leaching could modify the effective channel cross section and compromise reproducibility. The combined analysis therefore helps to define a more realistic operational window for the use of low-cost MSLA-printed resins in microfluidic prototyping.

Swelling percentages are shown in Tables S2, S3, and S4 in the Supporting Information corresponding to the measurements carried out after 1 h, 1 day, and 5 days, respectively. Figure 11 depicts a heatmap distribution of the swelling percentage per liquid. There, it is possible to see that the resins interact differently with the medium according to their nature. For instance, the Water-Wash + resin is the one swelling the most with aqueous media such as Milli-Q water and PBS, followed by the Tough Ultra and the ABS-like Pro 2. In other media, such as absolute ethanol, the Tough and ABS-like Pro 2 resins depict the maximum swellings after 5 days with values exceeding 15%. These two resins showcased noticeable swelling in isopropyl alcohol (IPA), reaching values of 8.54 and 4.29%, respectively. In general, the Standard V2 resin depicted small swelling in all 4 liquids, even with negative values such as the swelling in IPA. Finally, the High Clear resin exhibited considerable swelling in absolute ethanol (4.29% after 5 days), with minimal differences in the remaining scenarios.

**Figure 11:**
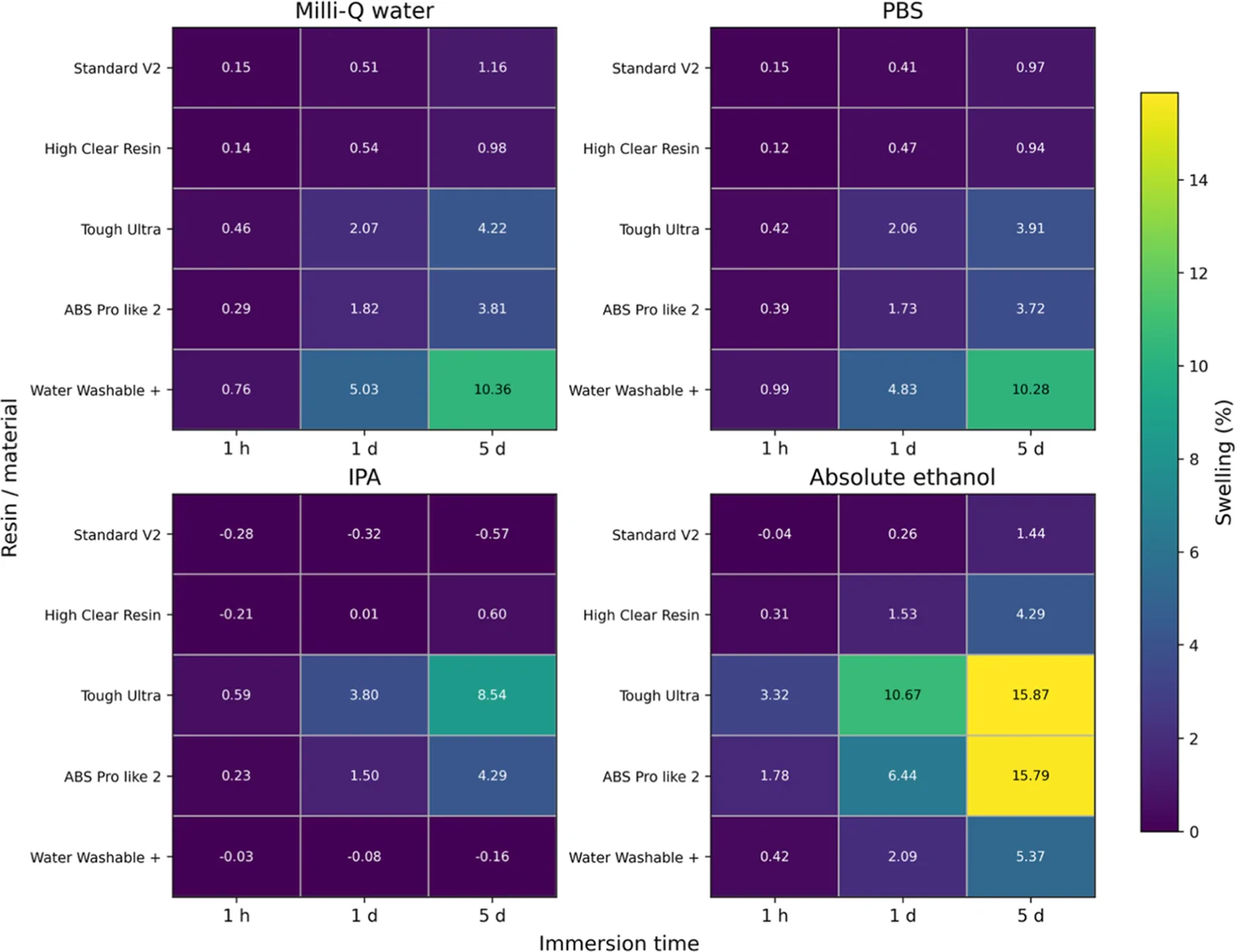
Swelling percentages for the five MSLA resins after 1 h, 1 day, and 5 days of immersion in four different liquids.

In other words, the swelling behavior of the resins was found to be dependent on the specific liquid the resin is in contact with. This fact is pivotal for long-term microfluidic assays, as increased swelling values can affect the structural stability of the chip, leading to the appearance of voids, cracks, and collapse of the channels.

## 4 Validation

The next step of this study was to create, print, and validate the mixing performance of different microfluidic devices. Three different systems were evaluated, the ones depicted in Figure 12: a simple Y-shaped microchannel, a Y-zigzagged channel, and a Y-shaped channel with microcylinders. Chips exhibit a rectangular profile of 1.5 × 0.75 mm. Microcylinders with a radius of r = 250 μm were selected.

**Figure 12:**
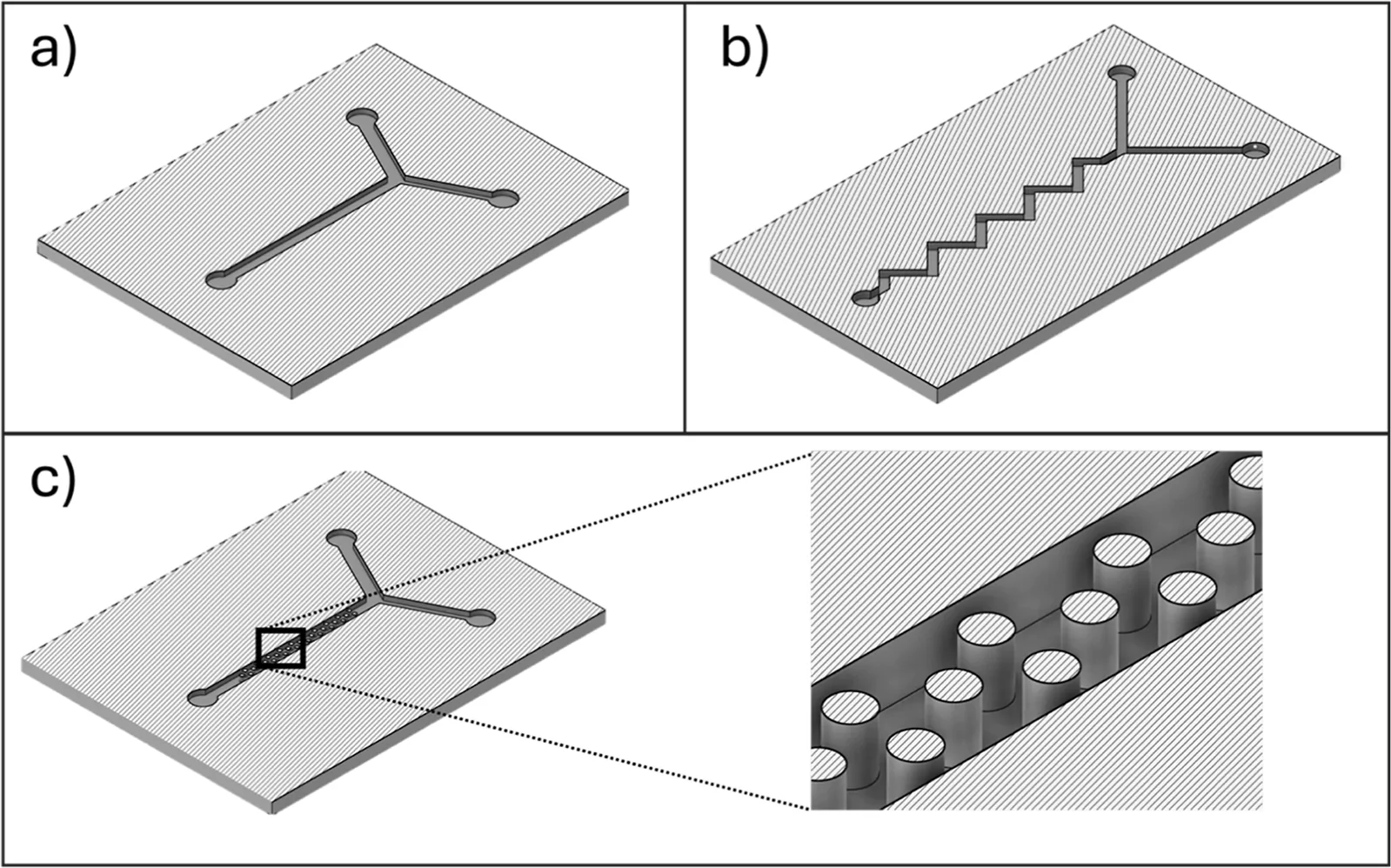
Microchannels chosen for the mixability tests. Chips with (a) Y-shape, (b) zigzag, and (c) microcylinders will be evaluated as mixers.

### 4.1 Computational Fluid Dynamics for Mixing Performance

The first step was to evaluate the mixability of the chips by means of computational fluid dynamics. Mixing histograms were obtained for each chip, as depicted in Figure 13. The water volume fraction was evaluated at the outlet of the Y-shaped chips, corresponding to a distance of *x* = 25 mm from the tip of the Y. That distance was taken as the reference for the chip with microcylinders and zigzagged, evaluating the water volume fraction at the mesh elements at that plane. Histograms were built with these data. The *x* axis corresponds to the water volume fraction. A value of 0.5 indicates perfect mixing, whereas values close to 0 and 1 correspond to the absence or the total presence of water. On the other hand, the *y* axis depicts the total number of counts of the volume fraction elements within the bin limits.

**Figure 13:**
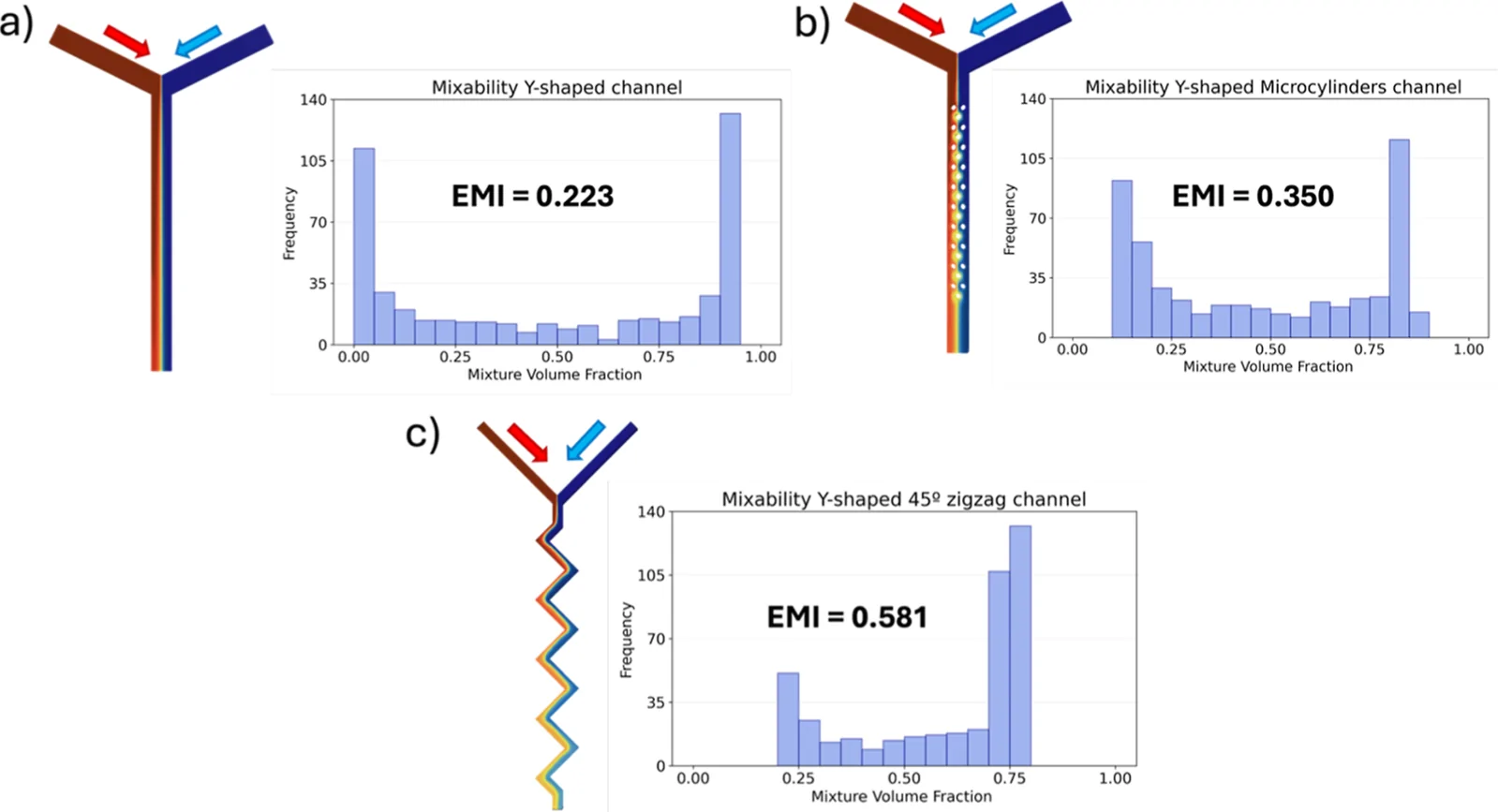
Passive scalar CFD simulations to evaluate the mixing capacity of three distinct chips. The chips exhibit (a) Y-shape, (b) microcylinders, and (c) zigzag. For each subpicture, the mixability histogram as well as its EMI value are depicted.

To facilitate the comprehension of the histograms and quantitatively assess the degree of mixing in the histograms, an Efficient Mixing Parameter (EMI) was defined as

6 $$EMI=\frac{Counts\;of\;water\;volume\;fraction\;between\;0.25-0.75}{Total\;number\;of\;counts}$$

EMI was calculated at the *x* = 25 mm plane section. The higher the value of EMI to 1.0, the higher the degree of mixing of the samples within the chip. The bounds 0.25–0.75 were selected as an operational concentration interval centered on the ideal, fully mixed value of 0.5. This criterion was used to identify regions where the scalar field no longer corresponded to the unmixed inlet stream.

The Y-shaped microfluidic chip is the one showcasing the lower EMI value due to the low Reynolds number common in this kind of system. This effect induces a laminar regime, where viscous forces dominate and layers of fluid are located parallel, resulting in minimal mixing. The only region where little mixing is noticeable corresponds to the interphase between the two distinct systems, as showcased in Video Material S1. Under this scenario, the EMI rises to a value of 0.223.

Moreover, the Y-shaped channel with microcylinders showcased a better performance than its counterpart, as seen by an EMI of 0.350. The presence of the microstructures fosters the appearance of recirculation throughout the channel, resulting in an operationally mixed region. Video Material S2 shows the effect of the cylinders on the fluid flow in the channel.

Finally, the zigzag channel mixing performance was analyzed at the plane at a distance of *x* = 25 mm from the Y tip. Under this scenario, the EMI rose to a maximum value of 0.581, depicting the suitability of this kind of structure for an operational mixing platform. The results are in accordance with the literature, where the zigzag configuration with an angle greater than or equal to 45° effectively improves mixing.^67,68^ The twisting sides turn the fluid flow to crush against the walls, encouraging the appearance of vorticity in the system (Dean vortices), as seen in Video Material S3.

In conclusion, CFD enabled us to compare the mixing performance of three distinct microchannels, showcasing that channels with microfeatures or with a zigzag shape promote an enhancement in the mixing performance. An experimental validation needs to be performed to contrast the aforementioned results.

### 4.2 Microfluidic Fluid Flow Assays for Mixing Validation

Experimental validation of the mixing performance was carried out. Three chips were printed in the Water-Wash + resin, given its higher transparency compared to the other resins, as seen in Figure 3. The total material cost for printing the three chips was 0.75 euros, demonstrating the cost effectiveness of the fabrication process. The chips are shown in Figure 14a.

**Figure 14:**
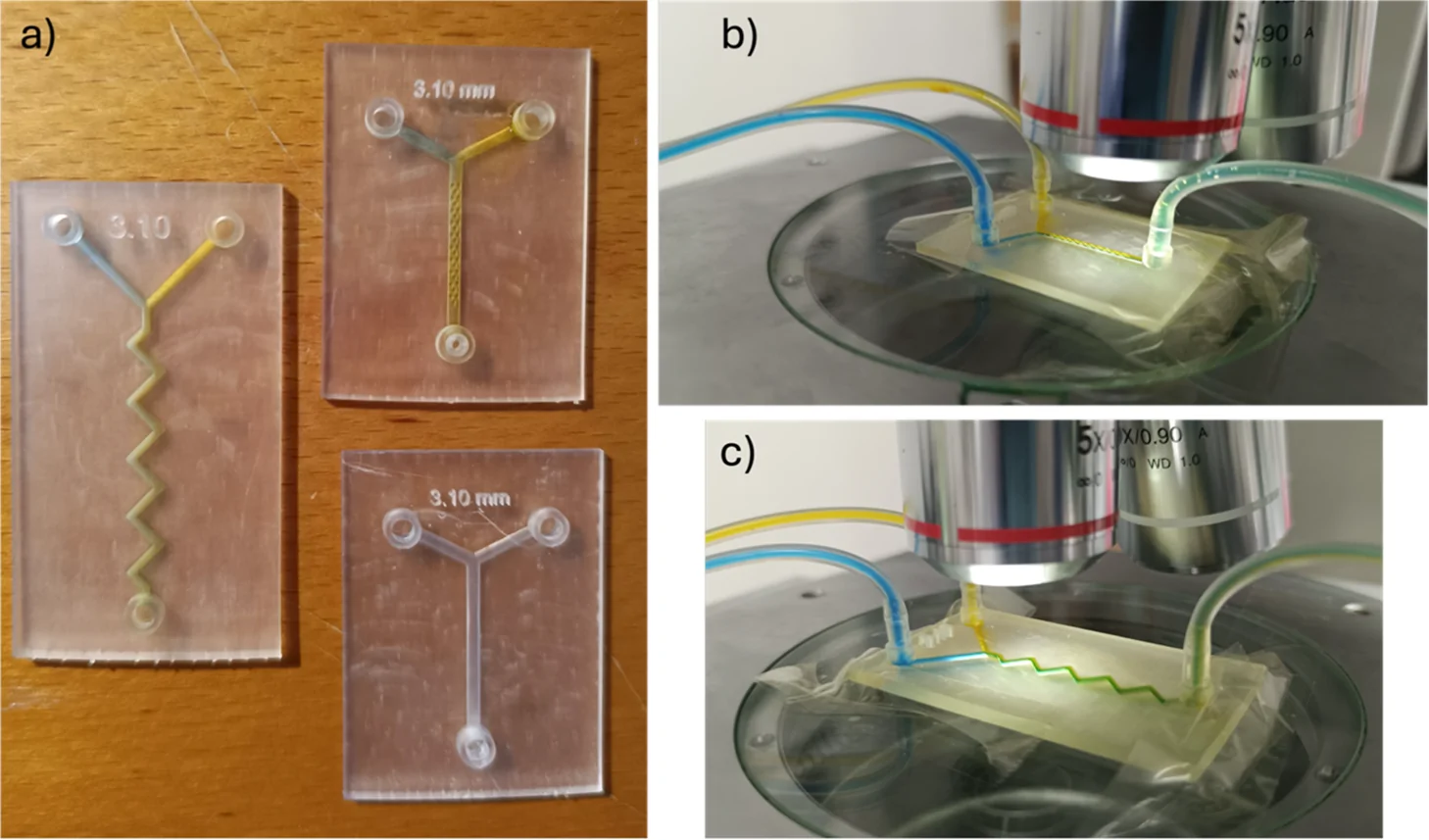
Experimental validation of the mixing performance for (a) three microfluidic chips printed with the water-washable resin. The chips were connected to the tubing system, and (b,c) optical microscopy images were obtained.

The experimental setup is depicted in Figure 14b,c. The peristaltic pump moves the fluid from the reservoirs to the two inlets. The optical microscope above the chip enables checking the mixing performance by collecting the resulting color mixture at the outlet. A final tube connected the outlet to a waste reservoir.

The fluid velocity at the inlet was set to 1 mm/s, and measurements of the final mixture color were performed, as shown in Figure 15. The upper row corresponds to the Y-shaped chip, the middle one collects the data from the chip featuring microstructures, and the lower one showcases the mixture from the zigzag chip.

**Figure 15:**
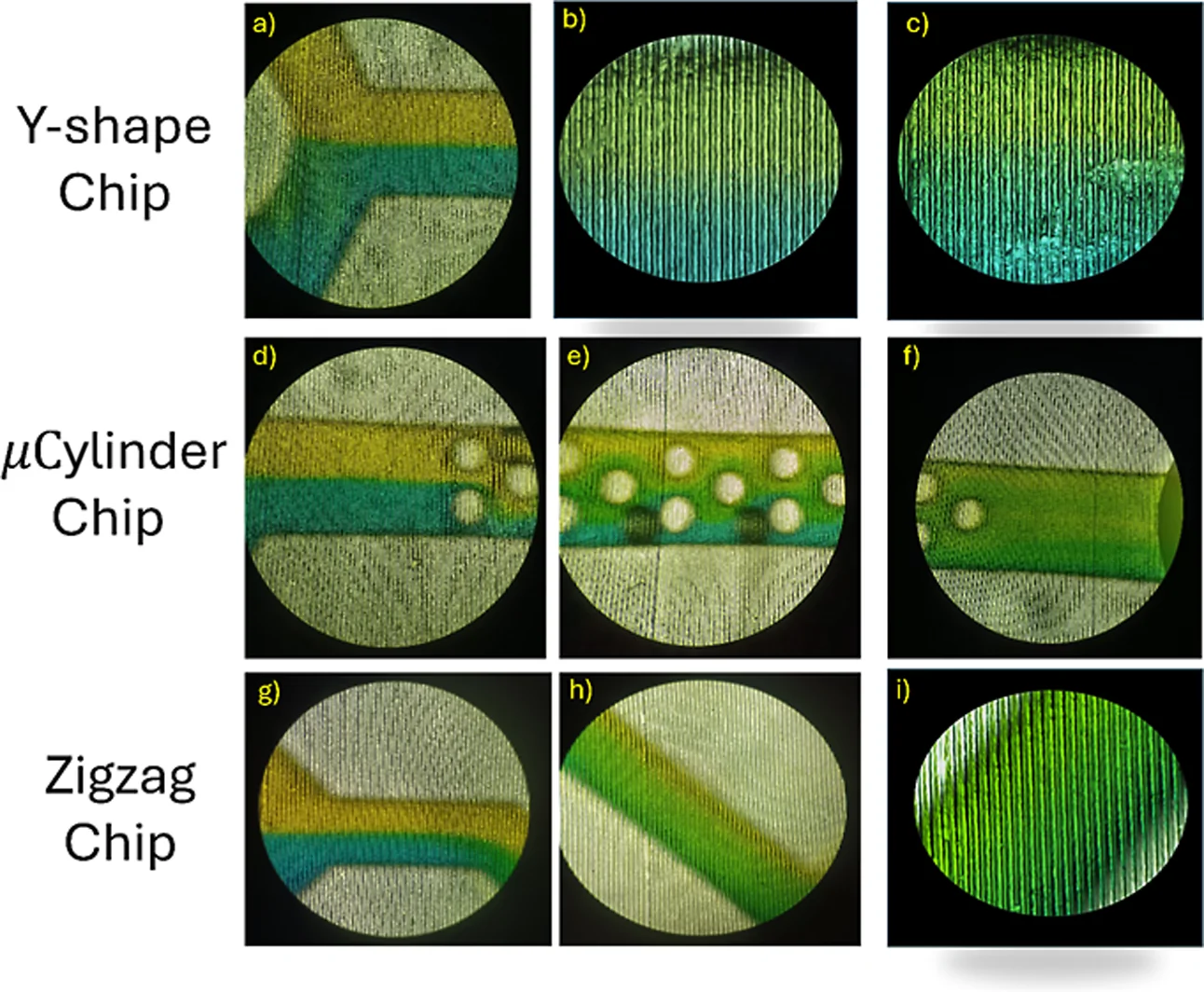
Mixing performance of the chips under a fluid assay test. The upper row depicts the Y-shape plain microfluidic chip in the (a) tip, (b) middle part, and (c) outlet of the Y. The middle row depicts the microcylinder chip, showcasing the two inks’ distribution in the (d) beginning, (e) middle, and (f) final parts of the chip. Finally, the row below reveals the mixability for the zigzag chip, showing the (g) beginning, (h) middle, and (i) final parts.

Starting from the top row, flow assay results are similar to the simulations. Laminar flow prevails, yielding the appearance of a neat interphase separating both inks. After the free propagation of the fluids, the outlet exhibits again a clear difference between the two inks. The final mixture is poor, as mentioned in the CFD simulation section. The videos Material S1 and S2 in the Supporting Information show the low mixability of the microfluidic platform in the inlet and outlet.

Moving to the middle row, mixability appears to be different compared with the plain Y-shaped channel. Under this scenario, the presence of microcylinders does have an effect on the circulation of the fluid, leading to a color variation along the chip. In the region close to the inlet, laminar flow predominates (Figure 15c). Nonetheless, once the fluids find the microcylinders, streamlines start moving around these obstacles, encouraging a subtle mixture along the chip. Finally, the outlet exhibits a greener color compared to its Y-shaped simple counterpart, proving that mixability has improved due to the presence of the microobstacles. These results are in accordance with the simulation, depicting that microcylinders are good candidates to break the laminar flow. Video Materials S3 and S4 showcase the difference in mixability prior to and after the propagation of the fluid through the set of microcylinders, illustrating how the sudden changes in streamline foster mixability.

The third row shows the mixability of the zigzag chip. Once again, laminar flow is present in the inlet of the system (Figure 15g). Once the fluids move along the chip, laminar flow is perturbed due to the successive collisions with the walls, resulting in an enhancement in the mixing proportional to the collisions performed. This effect results in the green-colored mixture in the middle (Figure 15h) and final (Figure 15i) parts of the channel. Video Material S5 depicts the propagation of both inks in the outlet and the resulting green color due to their combination.

These experimental results are in accordance with the CFD simulations: Y-shaped plain channels do not exhibit a correct mixability because of the stratified laminar flow. Conversely, the presence of obstacles in the micrometer size or irregular geometries does foster the mixability of the channel. The appearance of vorticity and the change in the direction of streamlines result in a mixing of the two dyes, resulting in a green final color. Nevertheless, the CFD approach showcased that mixability for the zigzag channel was much better than for the one with microcylinders, although experimental results were more alike than expected. This result could be due to many factors, including subtle variations in the dimensions and positions of the microcylinders compared to the simulated one. These variations could have enhanced the asymmetry of the system, resulting in better final mixing. On the other hand, small differences in the fluid velocity from both inlets may influence the manner in which the fluids are mixed, resulting in a different interaction between the fluids and the cylinders. Figure S12 shows the outlet for the 3 microfluidic systems, demonstrating the differences in mixability according to the case.

In conclusion, although the mixability for the channel featuring microfeatures and the zigzag one is similar, a pivotal result arises dispelling the conventional Y-shape microchannel resulted in systems with a better mixing performance. The printer was able to print not only three different chips with dimensions in the range of millimeters but also to recreate adequately a set of micrometric-size cylinders along the chip, showcasing the suitability of this affordable technology for out-of-care applications in the realm of microfluidics.

## 5 Limitations and Operational Window

The present results indicate that low-cost MSLA printing can be used as an accessible fabrication route for microfluidic prototyping and selected cell-related assays, but within a defined operating window which extends along optical transparency and working wavelengths, internal channel resolution, selective replication, and biological performance.

From an optical perspective, the transparent resins evaluated in this study provided sufficient transmission for direct visual inspection and conventional optical microscopy, although their use in optical assays must consider the material attenuation in depth, optical path length, surface roughness, and the reduced suitability of wavelengths close to and below the 405 nm photopolymerization wavelength. The five transparent resins depicted similar outcomes in terms of transparency, around 40% transmittance. Therefore, these materials should not be considered optically equivalent to glass, PDMS, or high-grade commercial optical polymers, especially for quantitative absorbance or fluorescence measurements without a prior calibration.

From a fabrication perspective, negative features and enclosed channels were more restrictive than positive surface features. This behavior is consistent with resin accumulation, incomplete drainage, and resin-dependent viscosity effects during the layer-by-layer printing and postprocessing steps. Under the printer settings and cleaning protocol used here, nominal internal channels of approximately 500 μm represented the lowest reliable channel dimension, whereas smaller channels showed reduced fidelity or incomplete clearance. The Standard, High Clear, and Water-Wash + resins were the ones depicting the best performance for the channels printed at the 500 μm resolution, especially when printed at angles such as 90° and 67.5°. A translation from the internal channel quarter-annulus structure toward a functional microfluidic chip was conducted on the Standard V2 resin, showcasing that channels with a nominal width slightly lower than 500 μm were possible to be printed in the 90° configuration. In other words, the simplified internal channel analysis can be successfully transferred to a lab-on-a-chip device.

From a structural point of view, swelling analysis enabled assessment of the structural stability of the samples during long periods of time for different solvents, both polar and nonpolar. For aqueous media, such as Milli-Q water or PBS, high swelling percentages were only obtained for the water-washable resin, yielding values above 10%. This fact is understandable given their different oligomeric nature, where residues after printing are released via washing with water. On the other hand, for nonpolar liquids such as isopropyl alcohol and absolute ethanol, the Tough and ABS-like Pro 2 resins exhibited notorious swelling, exceeding 15%. Finally, resins such as Standard V2 and High Clear showcased minimal swelling for almost all liquids after 5 days (a subtle swelling around 4% was found in absolute ethanol for the High Clear case), conferring longer structural stability and therefore being suitable for long-term lab-on-a-chip applications.

From a biological perspective, all tested resins showed non-cytotoxic behavior under the assays performed, with Standard V2 and High Clear showing the most favorable viability response. This improved performance was related to the static contact angle θ_*c*_ of the resins, where the ones depicting the strongest cell count exhibited the most hydrophilic-like behavior. This fact could explain why the application of plasma treatment enhances the viability of those resins depicting a hydrophobic nature. However, it is noteworthy to mention that biological compatibility remains dependent on the specific application and should be interpreted together with other factors such as the specific cell type under study or the swelling. In other terms, the platform is best suited for applications where the required optical performance, channel size, flow conditions, and biological endpoint fall within these experimentally validated boundaries.

This study establishes a practical operating window for low-cost MSLA-based microfluidic fabrication by defining the operational limits of the printing process and providing guidelines for selecting the most appropriate resin for specific applications, including biological assays, long-term structural stability, and high-resolution microchannel fabrication. As a practical design guide, Table 5 summarizes the operational windows of the five resins and identifies the most suitable material for each microfluidic application.

**Table 5:** Summary of the Performance of the Evaluated Resins for Different Applications, Highlighting the Most Suitable Candidate in Each Case

category	measured parameter	practical implication	best candidate
optical transparency	UV–Vis transmission	printed devices can be used for visual inspection and conventional microscopy	all five resins depicted similar maximum transmission. Water-Wash + was found to have slightly higher transmission
optical usability	spectral response around/above 405 nm	imaging and detection should avoid excitation and detection conditions strongly affected by resin absorption. Fluorescence is possible when the excitation wavelength is above 405 nm	all five resins showed a strong reduction in transmission below 405 nm
microfluidic channel fidelity	minimum reliable enclosed channel	designs should use channels above 500 μm for reliable fabrication with this printer and resin workflow	Standard V2, High Clear and Water-Wash + depicted better performance in printing angles close to 90°
cell viability	viability/cell count	resins should have enhanced cell viability for cell-dependent assays	Standard V2 and High Clear
dimensional reliability	positive vs negative feature dimensions	positive surface features are more reliable than negative ones due to resin accumulation and drainage direction	inward lines: Standard V2/High Clear
			inward cones: High Clear resin
			inward trap.: Standard V2/Water-Wash +
			outward lines: Tough/ABS Pro like 2
			outward cones: Tough/ABS Pro like 2
			outward trap.: Tough/High Clear
PDMS replicability	accuracy in the replication by optical inspection	the employment of low-cost resin molds to conduct soft lithography in PDMS	thermally cured PDMS: Tough ultra, ABS pro like 2 and Water-Wash +
			UV-light curable PDMS: ABS Pro like 2
wettability/Swelling	contact angle and swelling	fluid compatibility should be checked before long incubations or solvent exposure	hydrophobic: Tough Ultra, ABS-like Pro 2 and Water-Wash + resins
			hydrophilic: Standard V2 and High Clear resins
			Water-Wash + swells substantially in aqueous media (Milli-Q water and PBS)
			Tough ultra and ABS-like Pro 2 resins swell largely under contact in ethanol and isopropyl alcohol
			Standard V2 is robust in all 4 media
			High Clear is robust except for ethanol

## 6 Conclusions

The purpose of this study was to check the viability of employing low-cost MSLA 3D printers for research, especially to assess the suitability of the system for lab-on-a-chip applications. Different key elements were tested, such as the transmission spectra, surface resolution, replication capacity, and cytotoxicity. It was seen that most transparent resins exhibit a transmission of around 40%, which is enough to validate their performance as transparent microfluidic platforms by optical systems, such as optical microscopes. Fluorescence emerges as a possible characterization technique, although absorption and transmission wavelengths should be above 405 nm (absorption wavelength of the photopolymer from the resin).

Regarding surface characterization, a test on resolution was conducted. This depicts that, in general, negative structures’ resolution is lower than positive ones. In the negative configurations, subtle resin deposits are found in the direction the resin tends to slide during the layer-by-layer printing process, resulting in a reduction in the desired lengths and widths. The least differences in the ratio between theoretical and experimental dimensions were found in the cones, followed by the trapezoids and lines. Stability commitments were found in the lines exhibiting a smaller width compared to their heights, resulting in bending curves instead of straight lines. Regarding internal channels, limitations started to appear with diameters below 500 μm. The most viscous resins showed poorer performance than the more liquid ones due to an increase in the capillary forces and release times.

Regarding replicability, 3 out of the 5 resins were able to replicate thermally cured PDMS adequately. Among these 3 resins, the Tough was the one exhibiting a better performance, with a clear and neat replication of the z-step from the masters. This result differed from that of the UV-cured PDMS, where the ABS PRO was found to be the most effective resin for replica molding.

Cytotoxicity assessment was fundamental to showcasing the applicability of these resins to a biological realm. Cell viability was evaluated with direct methods, and it was found to be higher than 50% in adherent cells in all cases, with values close to 90% in resins such as the High Clear or the Standard. Similarly, the morphological analysis and cell count of circulating THP-1 cells at 72 h after seeding confirmed the biocompatibility of the different resins, being once more the High Clear and, mainly, the Standard resin particularly suitable for cell suspension cultures. These results, evaluated with two distinct cell cultures, open the door to employing this technology in biology-related branches within microfluidics, such as organ-on-a-chip approaches.

A final validation was performed using both computational and experimental approaches. The mixing performance of 3 distinct chips was evaluated. It was shown that the presence of microobstacles in the chip enhanced the mixability of the platform. The appearance of microvorticities in the system as well as the change of direction in the streamlines resulted in the interruption of the stratified and laminar flow to a more turbulent, mixing-fostering scenario. These results were also found in the zigzag channel, depicting that steep changes in the geometry of the chip did break laminar flow.

To sum up, the Anycubic Photon Mono M7 Pro printer was able to recreate a wide range of elements with proper reproducibility, evincing the suitability of these systems for such a broad field as microfluidics. The similarity in the results between simulations and experiments validates the performance of this system for lab-on-a-chip purposes, showcasing that affordable printers are suitable tools for this field. The democratization of research in microfluidics progressively turns into a reality.

## Supplementary Material













## Notes

Manufacturer recommended printing parameters, optical profilometry profiles of microstructures on the surface, images of THP-1 cells on plasma-treated commercial resins, cytotoxicity assays, contact angle, and swelling assays (PDF)
^•^CFD simulation Y-shape microchannel laminar flow in the inlet (MP4)
^•^CFD simulation Y-shape microchannel mixing phases in the outlet. Laminar flow hinders an appropriate mixing of both inks (MP4)
^•^CFD simulation fluid distribution from the mixer with microcylinders. Streamlines are affected by the presence of obstacles, fostering the mixing of both inks (MP4)
^•^Fluid distribution from the mixer with microcylinders in the outlet. The green color of the ink is an indicative of the mixing of the elements in both inlets (MP4)
^•^Fluid distribution from the zigzag mixer in the outlet. The abrupt change in direction gives rise to the appearance of vorticity in the system, resulting in a notorious degree of mixing (MP4)
