# Supplementary material for: Defining Operational Limits of Low-Cost MSLA 3D Printing for Microfluidic Systems: Resin Performance, Cell Compatibility, and Fluid Dynamics
Source: ACS Omega. 2026 Aug 12;11(33):49310–29. doi: 10.1021/acsomega.6c02218 (PMC13520512; doi:10.1021/acsomega.6c02218)
Supplement: Supplementary file 1 [file ao6c02218_si_001.pdf]

<u>SUPPORTING INFORMATION</u>

Defining Operational Limits of Low-Cost MSLA 3D Printing for Microfluidic Systems: Resin Performance, Cell Compatibility, and Fluid Dynamics

Yago Radziunas-Salinas[1,2], Bárbara Blanco-Fernández[2,3,4], Vanessa Valdiglesias[5], María Teresa Flores-Arias[1,2], Carmen Bao-Varela[1,2], Ana Isabel Gómez-Varela[1,2]*

[1]Photonics4Life Research Group, Applied Physics Department, Faculty of Physics 15782, Universidade de Santiago de Compostela, Campus Vida, Universidade de Santiago de Compostela, Spain

[2]Instituto de Materiais (iMATUS), Campus Vida, Universidade de Santiago de Compostela, Spain

[3]I+D Farma Group (GI-1645), Department of Pharmacology, Pharmacy and Pharmaceutical Technology, Universidade de Santiago de Compostela, E-15782 Santiago de Compostela, Spain

[4] Health Research Institute of Santiago de Compostela (IDIS), Complexo Hospitalario Universitario de Santiago de Compostela, Travesa da Choupana s/n., Santiago de Compostela 15706, Spain

[5]Universidade da Coruña, NanoToxGen Group, Centro Interdisciplinar de Química y Biología (CICA), Department of Biology, 15071 A Coruña, Spain

*Corresponding author: anaisabel.gomez@usc.es

Manufacturer recommended printing parameters

Table S1. Recommended printing parameters for the resins along the study.

Resin	Layer thickness (μm)	Exposure time (s)	Off time (s)	Bottom Exposure Time (s)	Bottom Layers	Anti-alias	Z Lift distance (mm)	Z lift Speed (mm/s)	Z retract distance (mm)	Cost (€)
**Water-Wash+**	50	1.8	2.0	25	5	1	8	6	6	25.99
**Standard V2**	50	1.8	0.5	25	5	1	8	6	6	21.99
**High Clear**	50	4	0.5	35	5	1	8	6	6	39.99
**ABS-like Pro 2**	50	1.8	1.0	25	5	1	8	6	6	28.99
**Tough Ultra**	50	1.8	2.0	30	5	1	8	6	6	38.99

Printing time

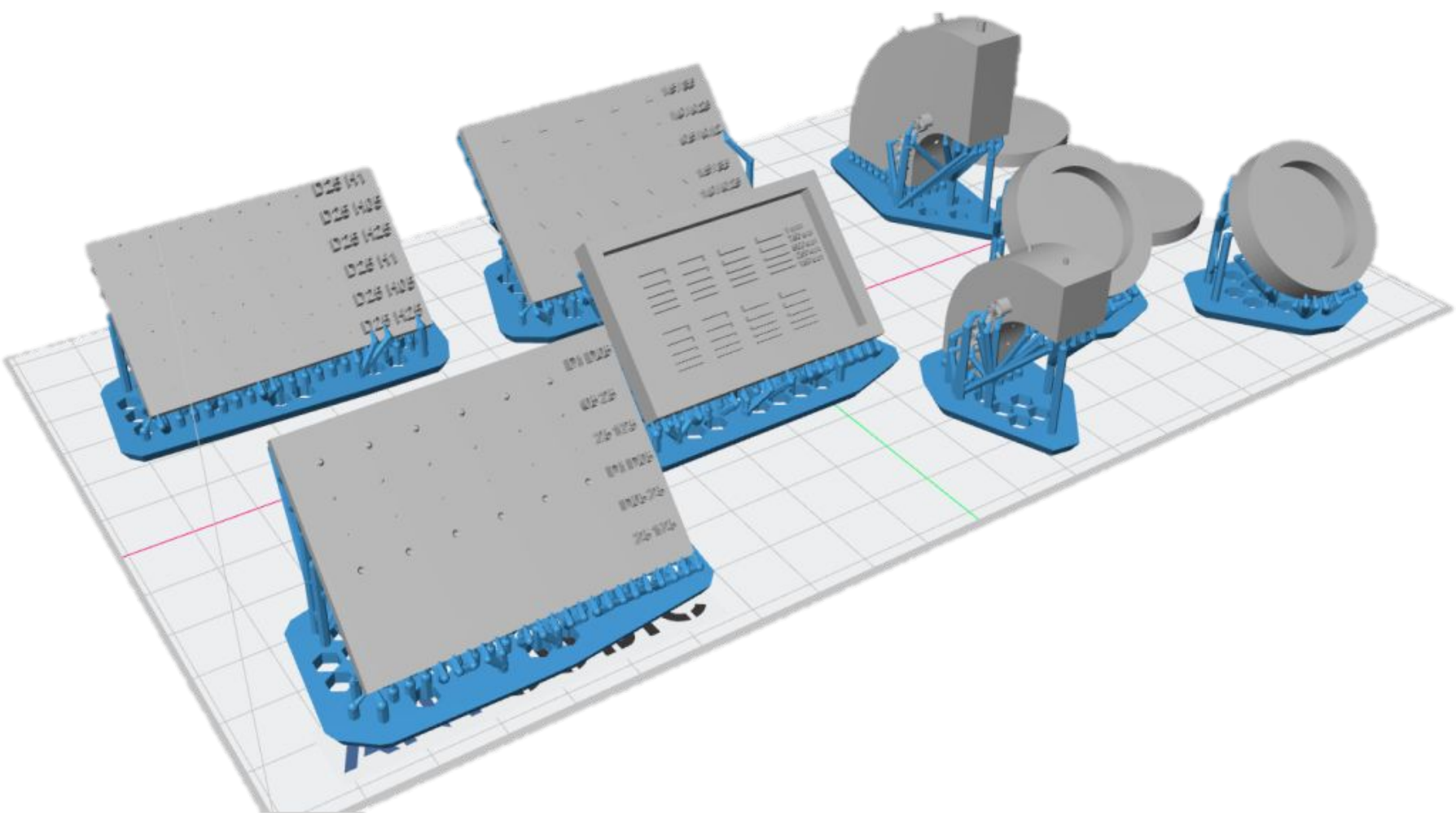

Figure S1. Printing time was evaluated for the following elements: plates featuring micro-lines, micro-cones, micro-trapezoids, internal channels, discs for transmission and biocompatibility assays.

Microstructures on the surface

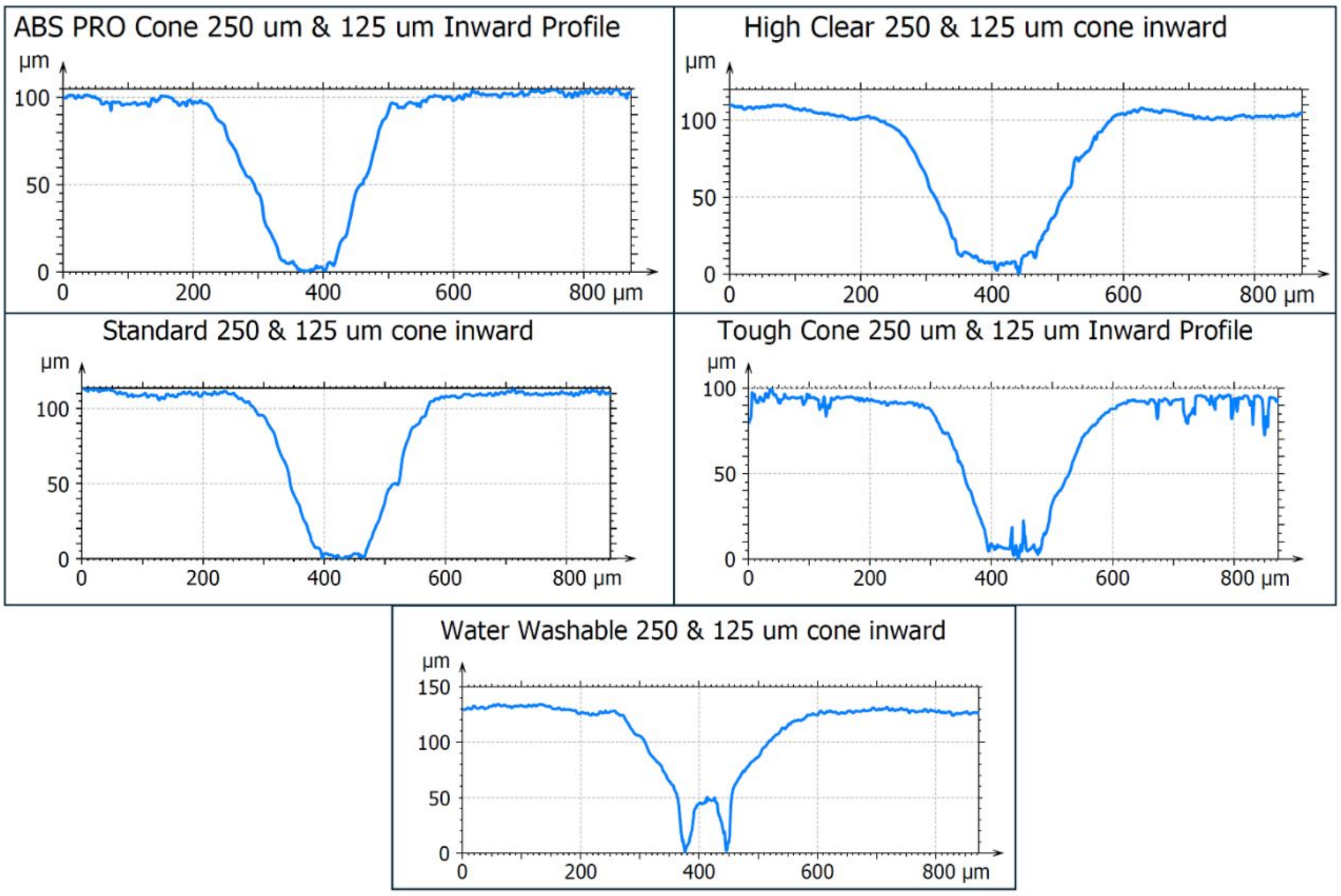

Figure S2. Negative cone structures profiles with D_1 = 250 μm and D_2 = 125 μm for the five resins of the study.

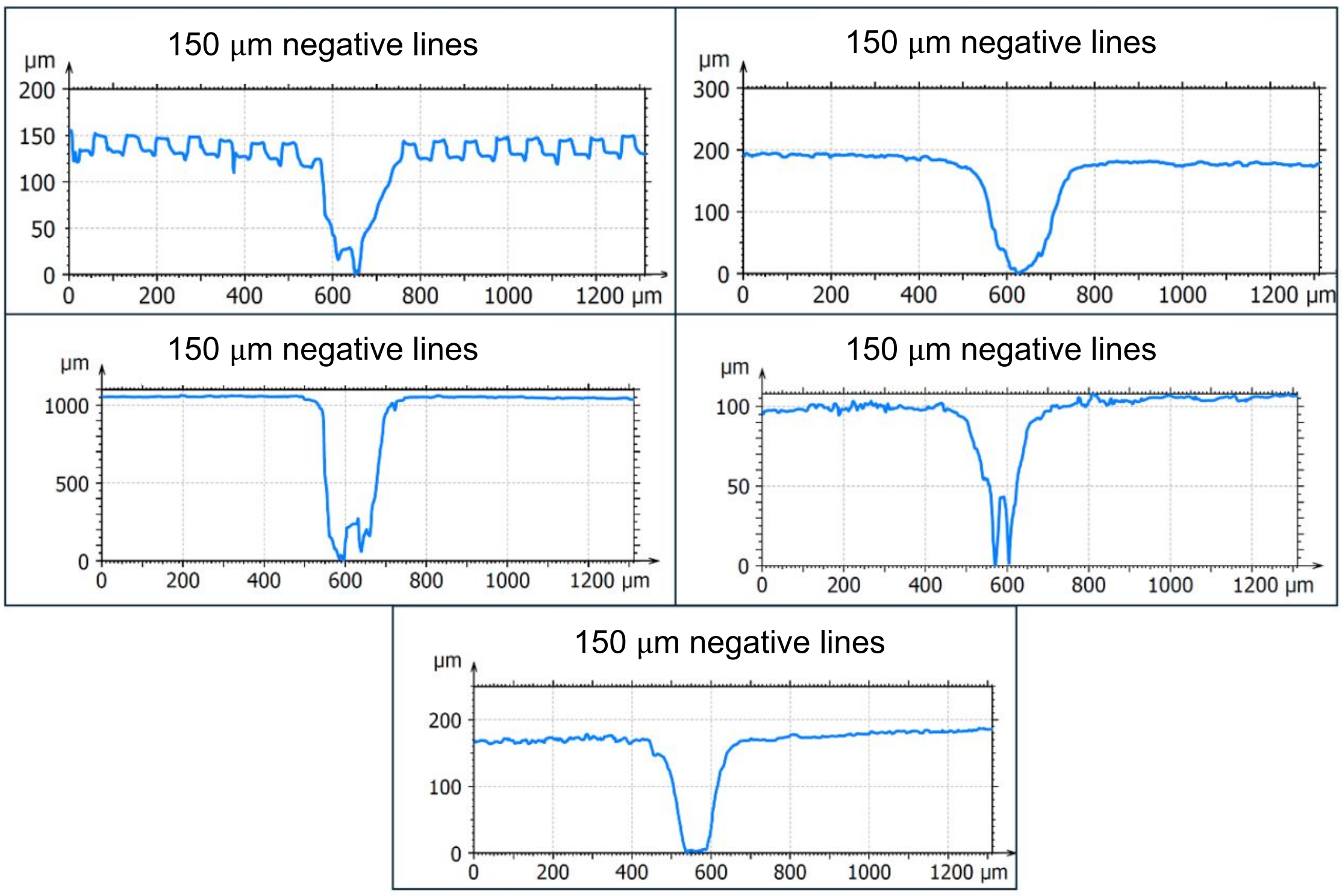

Figure S3. Negative line profiles with D = 150 μm.

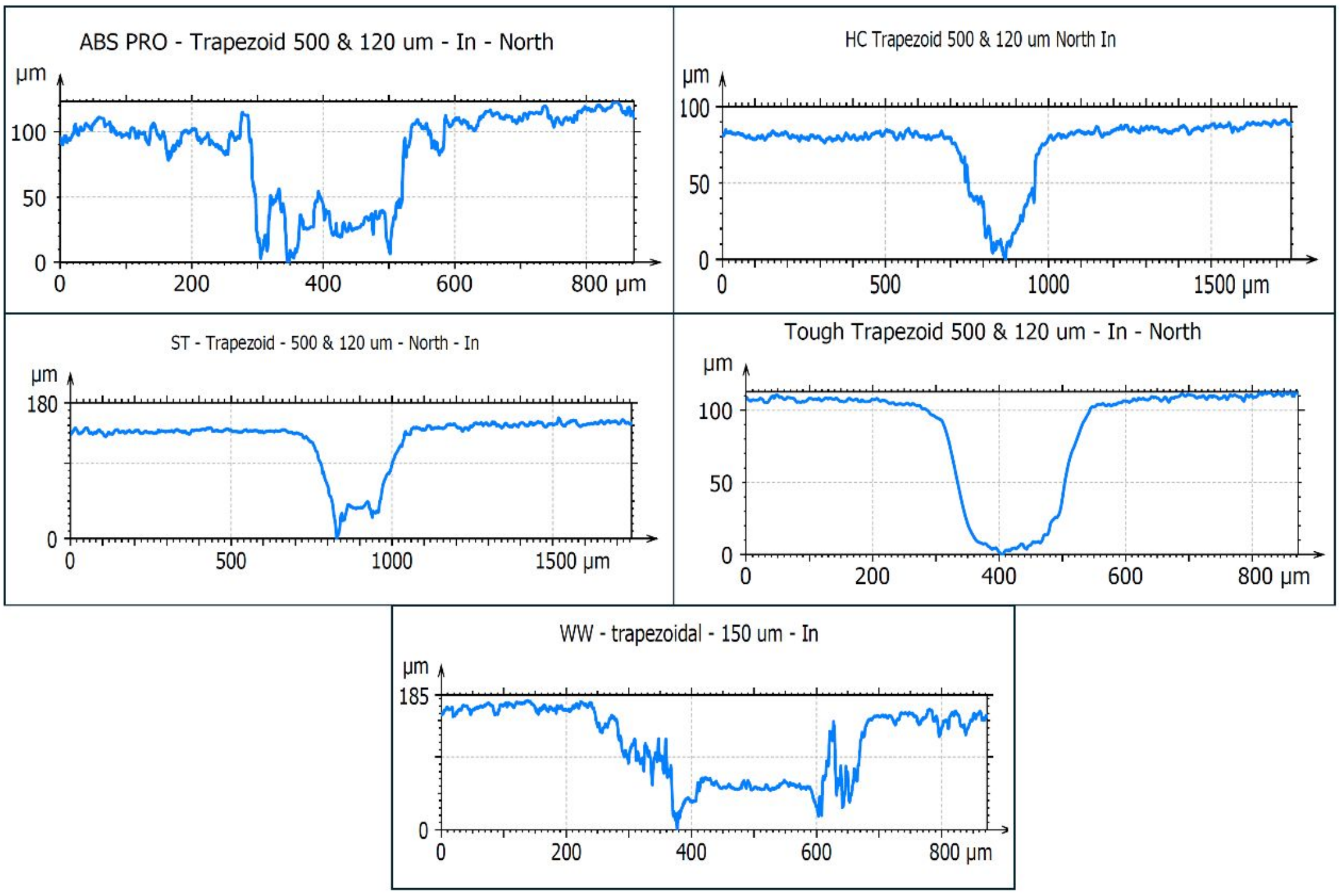

Figure S4. Negative trapezoidal profiles with L_1 = 120 μm.

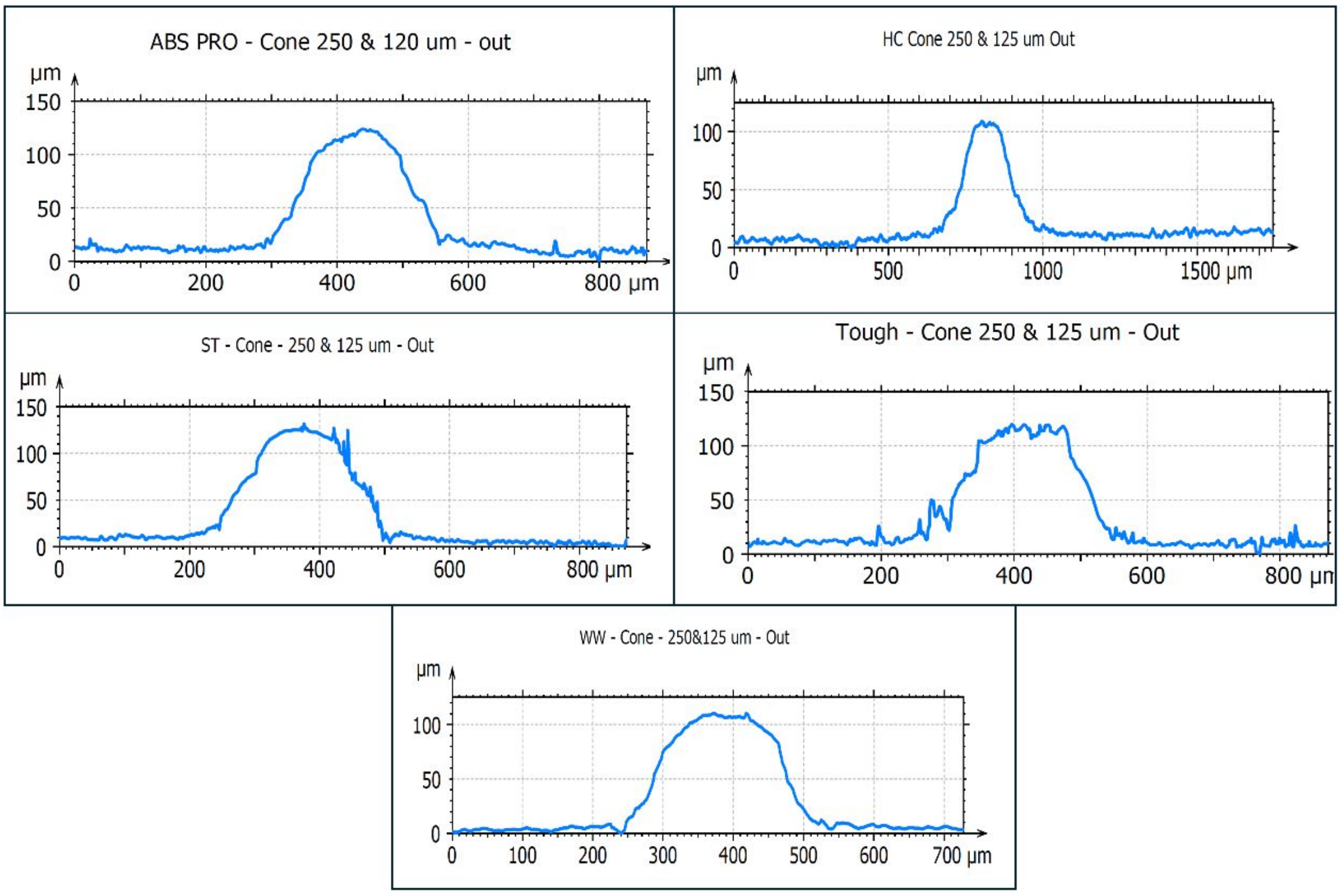

Figure S5. Positive cone profiles with D_1 = 250 μm and D_2 = 125 μm for the five resins of the study.

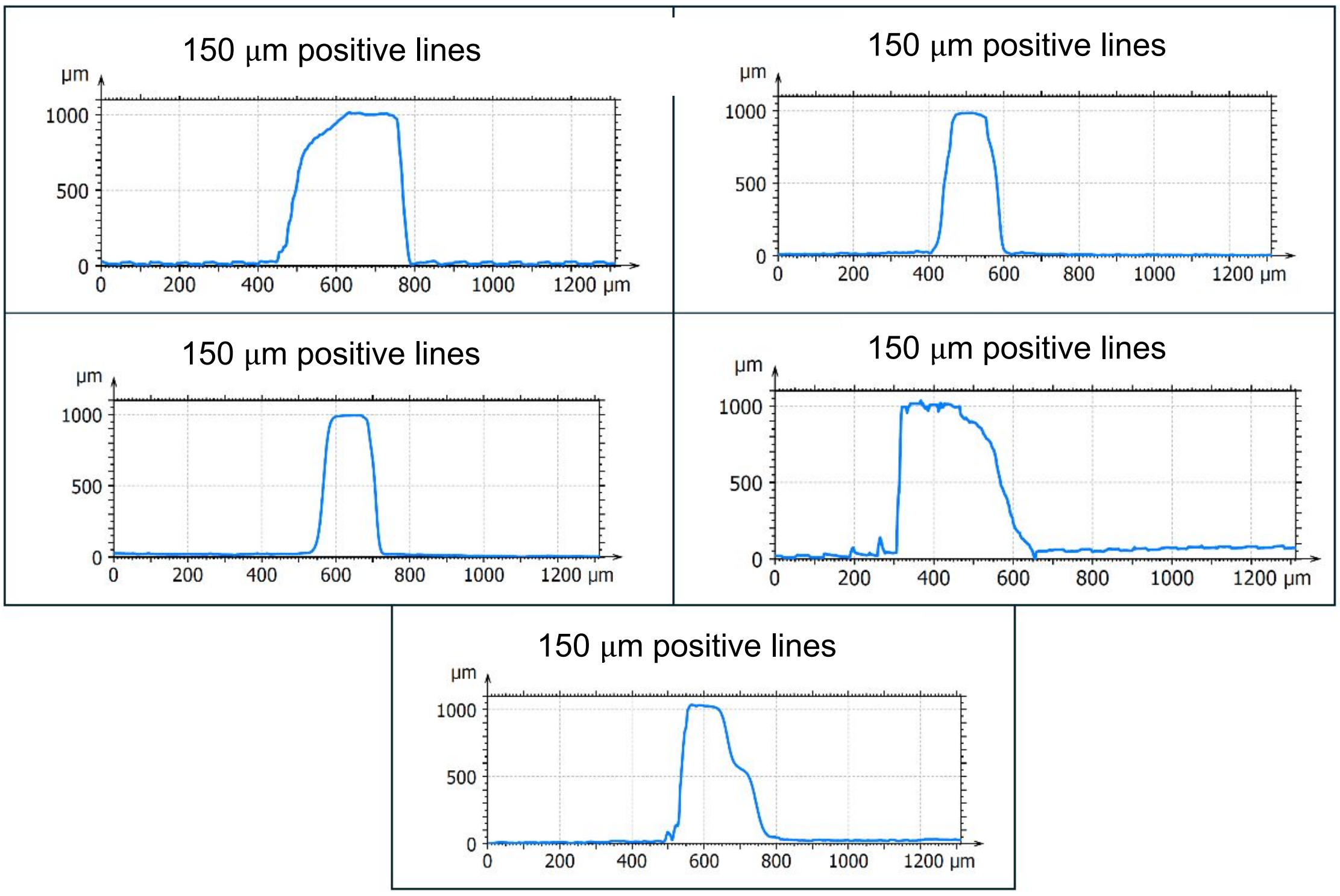

Figure S6. Positive line profiles with W = 150 μm.

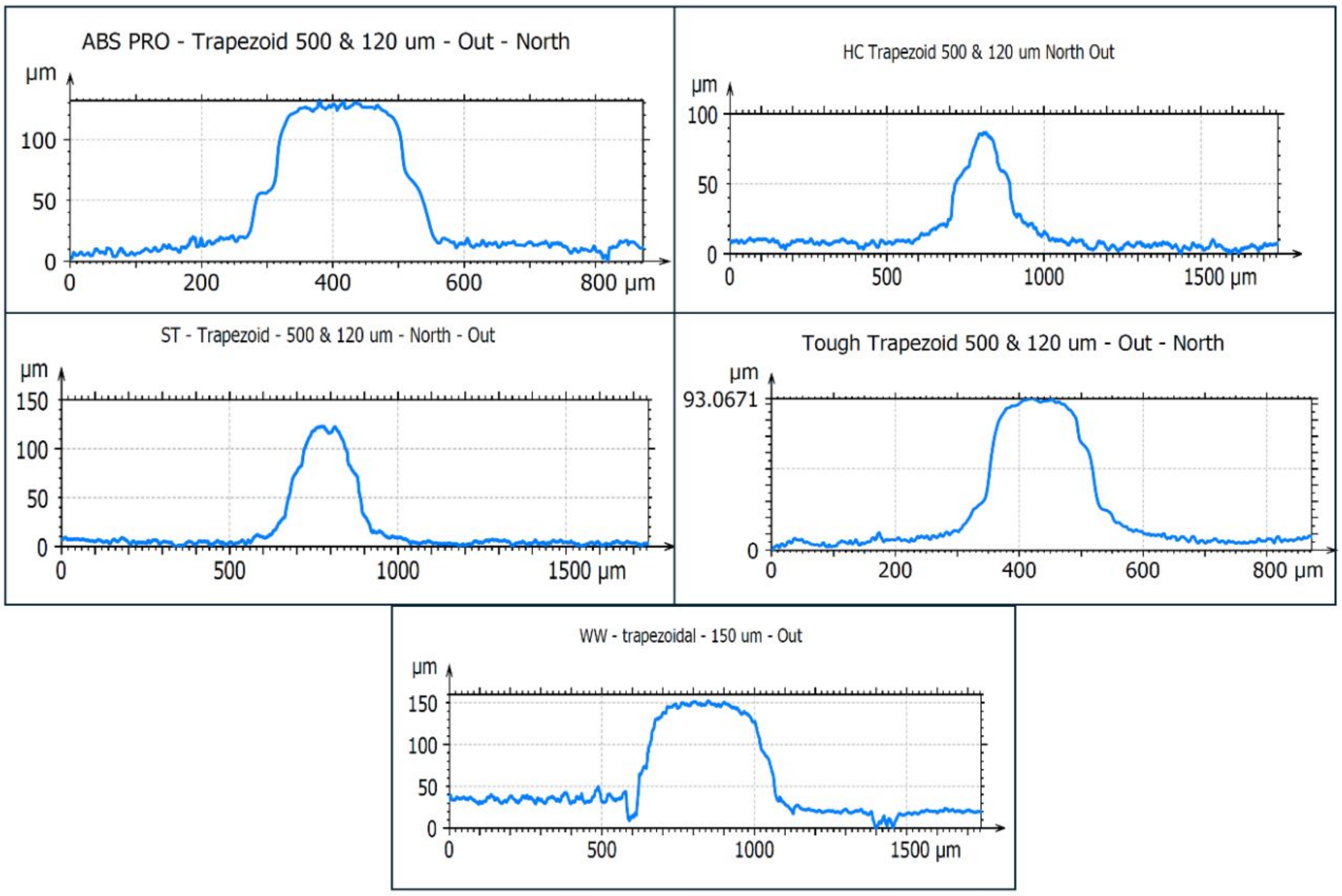

Figure S7. Positive trapezoidal profiles with L_1 = 150 μm.

Internal channels

A translation from the simplified internal channel assays towards a functional microfluidic device was conducted. Figure S8a) accounts for the different printing angles of the device, where the 90° configuration in the Standard V2 resin led to microfluidic chips (Figure S8b) with nominal width slightly below the 500 microns (Figure S8c).

The chip was subjected to a fluid flow assay (Figures S9a,b) under the same conditions as the other experiments conducted in this study, showing no leakage after 30 min of operation.

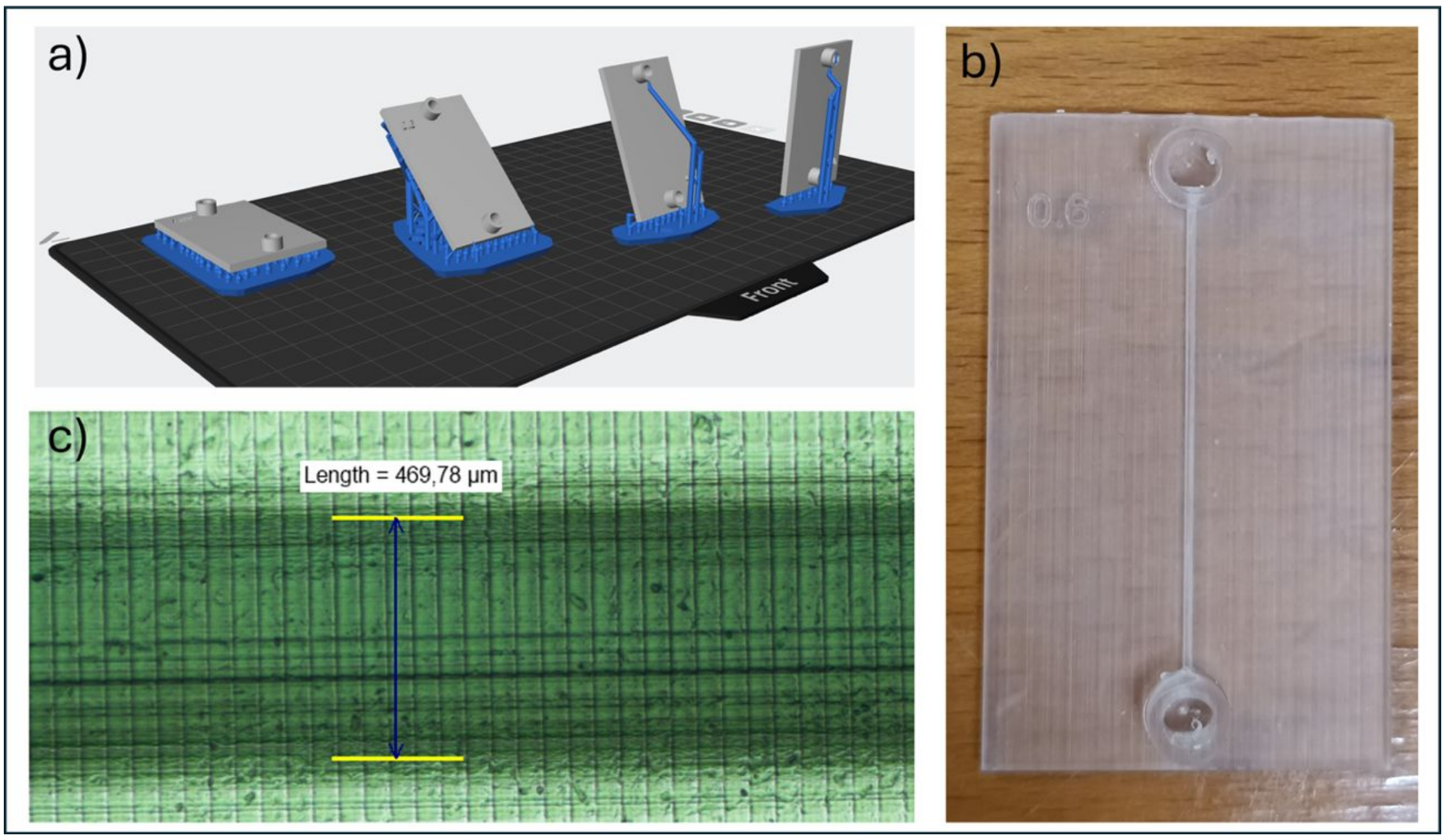

Figure S8. Evaluation of printing resolution using a functional microfluidic chip. a) Chips were printed at different orientations on the build platform. Only the 90° orientation enabled successful fabrication of a b) 600-µm-wide microchannel. (c) Optical microscopy measurements showed an actual channel width slightly below 500 µm.

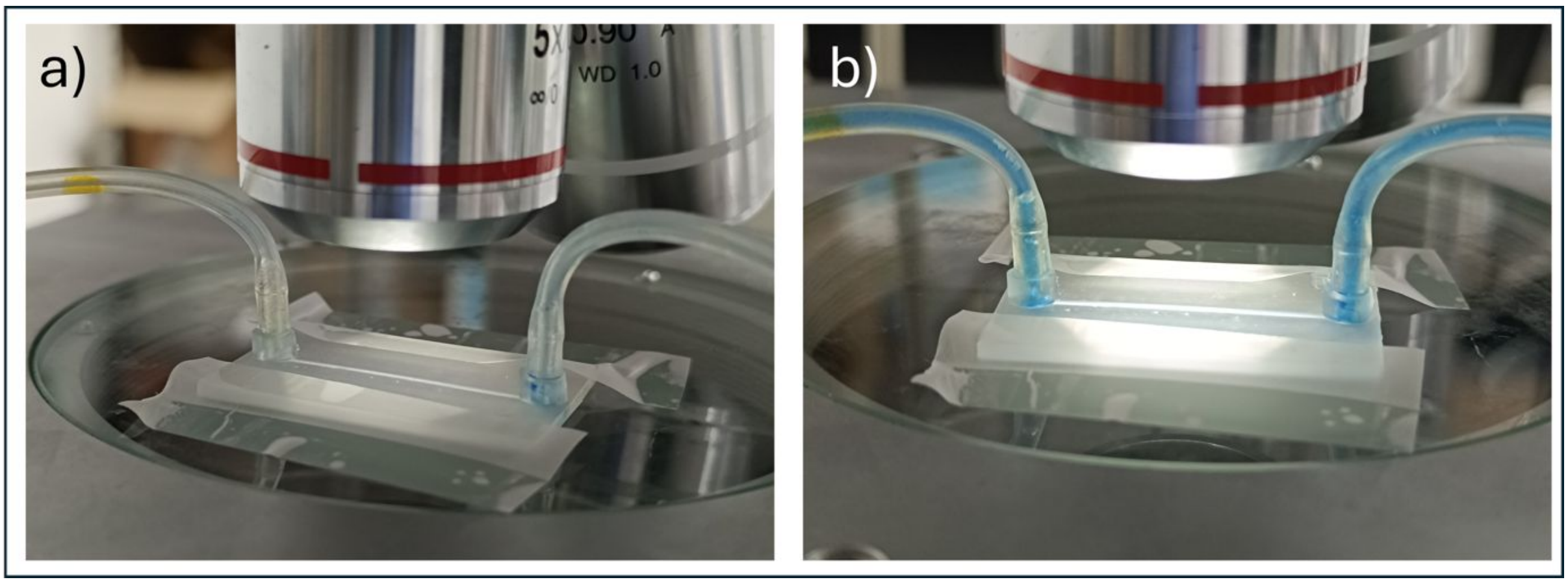

Figure S9. a) The microfluidic assay using the chip printed in the 90° orientation was performed under the same experimental conditions as the other flow experiments conducted in this study. b) The fluid flowed through the chip continuously for 30 min without any visible leakage.

Cytotoxicity assays

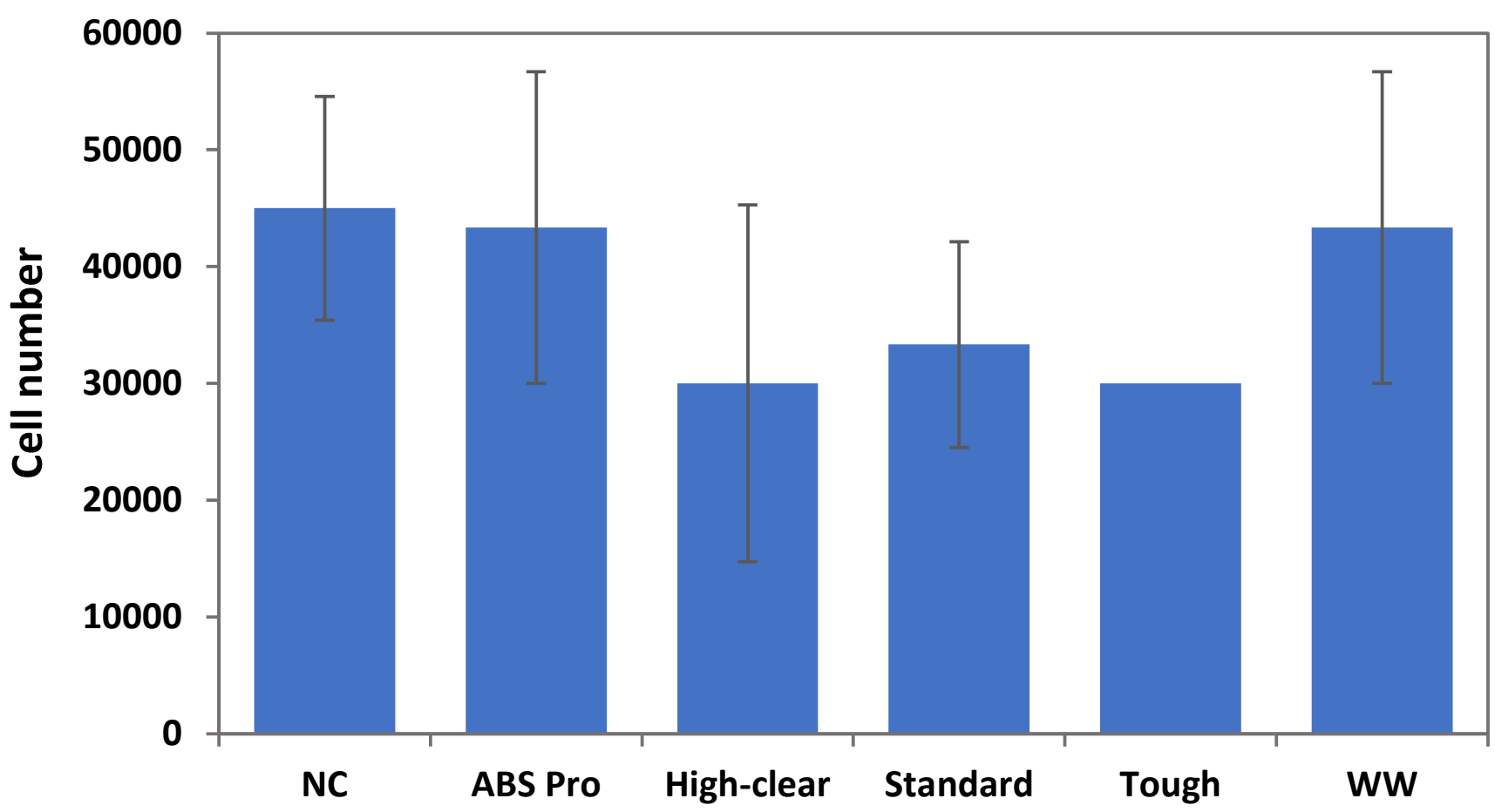

Figure S10. Cell count of THP-1 cells cultured on plasma-treated resins for 72h.

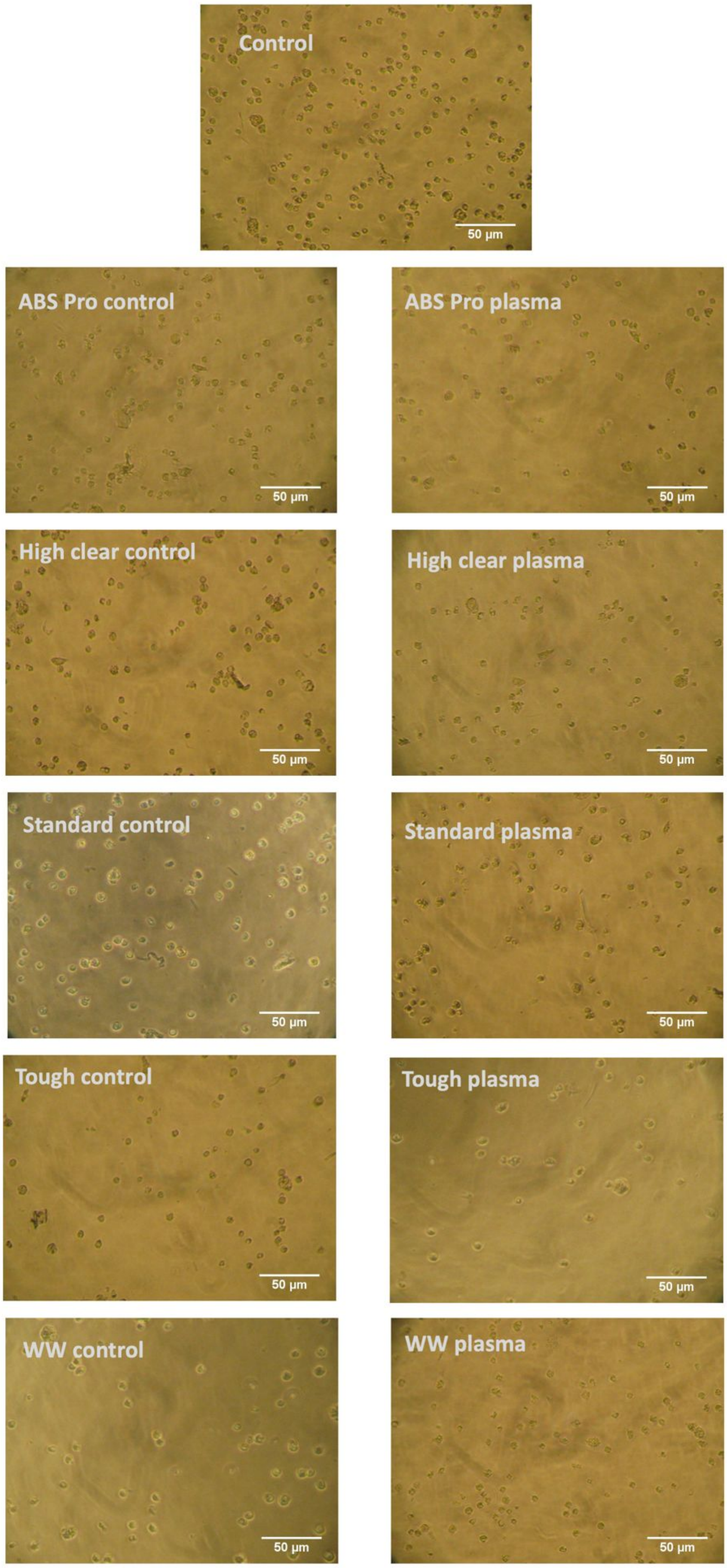

Figure S11. Phase contrast optical microscope images of THP-1 cells taken using a 10x magnification objective.

Contact angle and swelling assays

Table S2. Swelling percentage (mean ± SD) after 1 hour immersion.

Material	Milli-Q water	PBS	IPA	Absolute ethanol
Standard V2	0.15 ± 0.10	0.151 ± 0.011	-0.28 ± 0.13	-0.04 ± 0.11
High Clear Resin	0.142 ± 0.070	0.122 ± 0.043	-0.21 ± 0.24	0.313 ± 0.047
Tough Ultra	0.46 ± 0.23	0.422 ± 0.082	0.59 ± 0.24	3.32 ± 0.65
ABS Pro like 2	0.29 ± 0.12	0.391 ± 0.070	0.238 ± 0.038	1.78 ± 0.20
Water Washable +	0.763 ± 0.072	0.99 ± 0.11	-0.03 ± 0.10	0.42 ± 0.10

Table S3. Swelling percentage (mean ± SD) after 1 day immersion.

Material	Milli-Q water	PBS	IPA	Absolute ethanol
Standard V2	0.512 ± 0.031	0.412 ± 0.012	-0.32 ± 0.060	0.263 ± 0.011
High Clear Resin	0.54 ± 0.12	0.474 ± 0.014	0.01 ± 0.15	1.535 ± 0.053
Tough Ultra	2.07 ± 0.26	2.06 ± 0.31	3.80 ± 0.81	10.67 ± 1.88
ABS Pro like 2	1.822 ± 0.088	1.729 ± 0.036	1.50 ± 0.19	6.44 ± 0.57
Water Washable +	5.03 ± 0.22	4.83 ± 0.20	-0.08 ± 0.13	2.09 ± 0.19

Table S4. Swelling percentage (mean ± SD) after 5 days immersion.

Material	Milli-Q water	PBS	IPA	Absolute ethanol
Standard V2	1.162 ± 0.071	0.97 ± 0.26	-0.57 ± 0.16	1.44 ± 0.77
High Clear Resin	0.98 ± 0.23	0.94 ± 0.092	0.604 ± 0.014	4.298 ± 0.072
Tough Ultra	4.22 ± 0.42	3.91 ± 0.63	8.54 ± 1.77	15.87 ± 1.36
ABS Pro like 2	3.813 ± 0.093	3.721 ± 0.091	4.29 ± 0.83	15.79 ± 1.45
Water Washable +	10.36 ± 0.16	10.28 ± 0.76	-0.16 ± 0.52	5.37 ± 0.54

Microfluidic fluid flow assays for mixing validation

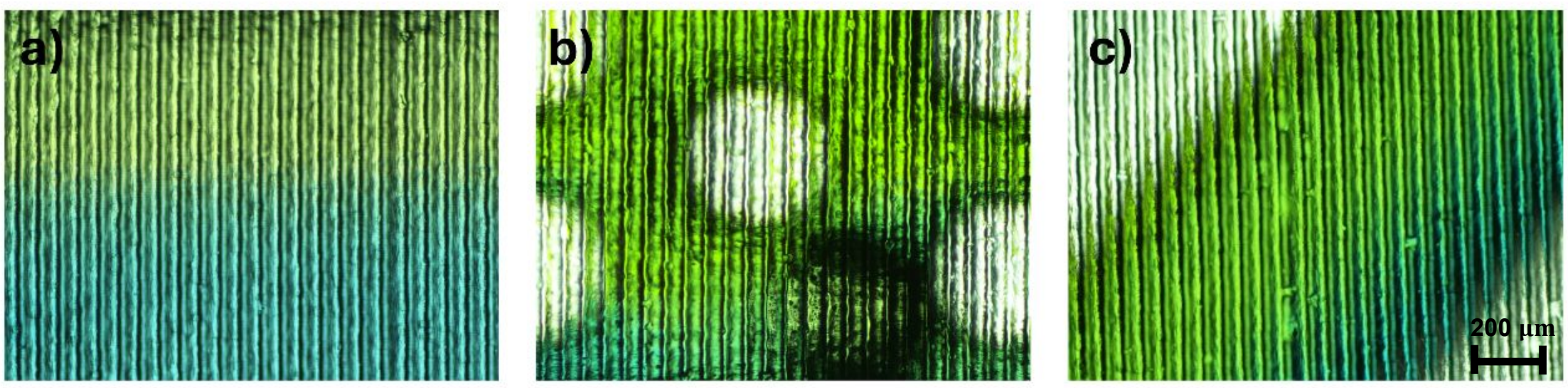

Figure S12. Optical microscopy images for the three micromixers, including a a) Y-shape channel, b) set of micro-cylinders and c) zigzag channel. Scale is 200 µm.
